# Pi-Dan-Jian-Qing Decoction Ameliorates Type 2 Diabetes Mellitus Through Regulating the Gut Microbiota and Serum Metabolism

**DOI:** 10.3389/fcimb.2021.748872

**Published:** 2021-12-06

**Authors:** Xuehua Xie, Jiabao Liao, Yuanliang Ai, Jinmei Gao, Jie Zhao, Fei Qu, Chao Xu, Zhaiyi Zhang, Weibo Wen, Huantian Cui, Hongwu Wang

**Affiliations:** ^1^ First College of Clinical Medicine, Nanjing University of Traditional Chinese Medicine, Jiangsu, China; ^2^ Department of Endocrinology, Yunnan Provincial Hospital of Chinese Medicine, Yunnan, China; ^3^ Department of Emergency, Jiaxing Hospital of Traditional Chinese Medicine, Zhejiang, China; ^4^ Jiaxing Key Laboratory of Diabetic Angiopathy Research, Jiaxing Hospital of Traditional Chinese Medicine, Zhejiang, China; ^5^ Department of Orthopedics, Kunming Municipal Hospital of Traditional Chinese Medicine, Yunnan, China; ^6^ Department of Rehabilitation, Fujian People’s Hospital of Traditional Chinese Medicine, Fujian, China; ^7^ College of Integrated Chinese and Western Medicine, Tianjin University of Traditional Chinese Medicine, Tianjin, China; ^8^ Shandong Provincial Key Laboratory of Animal Cell and Developmental Biology, School of Life Sciences, Shandong University, Shandong, China; ^9^ College of Traditional Chinese Medicine, Tianjin University of Traditional Chinese Medicine, Tianjin, China

**Keywords:** type 2 diabetes mellitus, Pi-Dan-Jian-Qing decoction, gut microbiota, tryptophan metabolism, histamine metabolism, TCA cycle

## Abstract

Pi-Dan-Jian-Qing decoction (PDJQ) can been used in the treatment of type 2 diabetes mellitus (T2DM) in clinic. However, the protective mechanisms of PDJQ on T2DM remain unknown. Recent studies have shown that the changes in gut microbiota could affect the host metabolism and contribute to progression of T2DM. In this study, we first investigated the therapeutic effects of PDJQ on T2DM rats. 16S rRNA sequencing and untargeted metabolomics analyses were used to investigate the mechanisms of action of PDJQ in the treatment of T2DM. Our results showed that PDJQ treatment could improve the hyperglycemia, hyperlipidemia, insulin resistance (IR) and pathological changes of liver, pancreas, kidney, and colon in T2DM rats. PDJQ could also decrease the levels of pro-inflammatory cytokines and inhibit the oxidative stress. 16S rRNA sequencing showed that PDJQ could decrease the *Firmicutes/Bacteroidetes* (*F* to *B*) ratio at the phylum level. At the genus level, PDJQ could increase the relative abundances of *Lactobacillus*, *Blautia, Bacteroides, Desulfovibrio* and *Akkermansia* and decrease the relative abundance of *Prevotella*. Serum untargeted metabolomics analysis showed that PDJQ could regulate tryptophan metabolism, histidine metabolism, tricarboxylic acid (TCA) cycle, phenylalanine, tyrosine and tryptophan biosynthesis and tyrosine metabolism pathways. Correlation analysis indicated that the modulatory effects of PDJQ on the tryptophan metabolism, histidine metabolism and TCA cycle pathways were related to alterations in the abundance of *Lactobacillus*, *Bacteroides* and *Akkermansia*. In conclusion, our study revealed the various ameliorative effects of PDJQ on T2DM, including improving the liver and kidney functions and alleviating the hyperglycemia, hyperlipidemia, IR, pathological changes, oxidative stress and inflammatory response. The mechanisms of PDJQ on T2DM are likely linked to an improvement in the dysbiosis of gut microbiota and modulation of tryptophan metabolism, histamine metabolism, and the TCA cycle.

## Introduction

Type 2 diabetes mellitus (T2DM) is a complex endocrine disease caused by a combination of environmental and genetic factors, leading to islet failure or insufficient insulin action. According to data from the World Health Organization, 415 million patients were suffering from T2DM in 2015 and this number will increase to 642 million by 2040 (Rivera-Mancía et al., 2015). The etiology of T2DM has unfortunately not been fully elucidated, and the lack of effective treatment is a major health challenge globally.

The human intestine is a complex micro-ecosystem. The bacteria residing in the intestine are large in number and diverse, and have important roles in regulating host metabolism. Under physiological conditions, the intestinal microbiota can maintain the homeostasis of host glucose and lipid metabolism by digesting and absorbing food and producing metabolites (Ortega et al., 2020). Unhealthy dietary habits can destroy certain species of intestinal bacteria and affect the body’s metabolism, which may eventually lead to the occurrence of T2DM. Compared with the status in healthy people, the abundances of many probiotic bacteria including *Lactobacillus* and *Akkermansia* are decreased in the intestine of T2DM patients, while some harmful bacteria such as *Enterobacteriaceae* are increased (Tanase et al., 2020). Furthermore, the abnormal levels of metabolites caused by intestinal microbiota disorder are closely related to inflammatory reactions, insulin resistance (IR), as well as the disorders of glucose and lipid metabolism (Chávez-Carbajal et al., 2020). Probiotic interventions, including with *Roseburia*, *Bacteroides*, *Akkermansia*, and *Lactobacillus*, can help improve glucose metabolism disorders and restore insulin sensitivity (Huda et al., 2021). Study have demonstrated that metformin could regulate the abundances of *Bacteroidetes*, *Escherichia*, and other microbiota, thus maintaining the integrity of the intestinal barrier, promoting the production of short-chain fatty acids (SCFAs), and regulating bile acid metabolism, thereby showing hypoglycemic effects (Zhang and Hu, 2020).

Traditional Chinese medicine (TCM) has been used to treat T2DM for thousands of years. Meta-analysis indicated that TCM could reduce the risk of progression to T2DM and increase the possibility of regression toward normoglycemia (Pang et al., 2017). It is important to investigate the mechanism of action of TCM in the treatment of T2DM, which can help not only to reveal the implications of its action, but also provide a scientific basis for new drug discovery. Regulation of the intestinal microbiota has become an important target of TCM for the treatment of T2DM. Isomaltulose can increase the abundance of *Actinobacteria*, *Faecalibacterium*, *Blautia*, and *Phascolarctobacterium*, while it decreases the abundance of *Patescibacteria* in the gastrointestinal (GI) tract of T2DM rats, thereby regulating the metabolism of lipids, carbohydrates, and bile acids (Yang et al., 2021). Oxymatrine has been shown to help reduce the abundances of *Phascolarctobacterium*, *Roseburia*, and *Prevotella* microbiota in the GI tract and increase the abundances of *Bacteroidetes*, *Ruminococcus*, and *Streptococcus* microbiota to regulate glucose metabolism, lipid metabolism, and carbohydrate metabolism, and to improve pathoglycemia and dyslipidemia in T2DM rats (Shao et al., 2020). *Ganoderma lucidum* polysaccharides could reduce the abundances of harmful bacteria, such as *Ruminococcus*, *Corynebactrium*, and *Proteus*, in the GI tract of T2DM rats, increase the abundances of *Blautia*, *Dehalobacterium*, *Parabacteroides*, and *Bacteroides*, and restore the disordered metabolism of amino acids and carbohydrates in T2DM rats (Chen et al., 2020). By increasing the abundance of *Parabacteroides*, *Blautia*, and *Akkermansia* microbiota and decreasing the abundance of *Aerococcus*, *Staphylococcus*, and *Corynebacterium* microbiota in the GI tract, Huang-Lian-Jie-Du decoction helped improve lipid metabolism and bile acid metabolism in T2DM rats, and improve IR (Chen et al., 2018).

Pi-Dan-Jian-Qing decoction (PDJQ), composed of Astragalus mongholicus Bunge, Pseudostellaria heterophylla (Miq.) Pax, Atractylodes lancea (Thunb.) DC., Scrophularia ningpoensis Hemsl., Coptis chinensis Franch., Scutellaria baicalensis Georgi, Pueraria montana var. lobata (Willd.) Maesen & S.M.Almeida ex Sanjappa & Predeep, Potentilla discolor Bunge, Litchi chinensis Sonn. and Salvia miltiorrhiza Bunge, has been used to treat T2DM in Yunnan Provincial Hospital of Chinese Medicine, Kunming Municipal Hospital of Traditional Chinese Medicine and Jiaxing Hospital of Traditional Chinese Medicine for more than 10 years. To date, over 1000 T2DM patients have received the treatment of PDJQ. According to our clinical study, treatment with PDJQ in combination with metformin significantly decreased the fasting blood glucose (FBG) and postprandial blood glucose levels and improved the clinical outcomes such as polydipsia and fatigue in T2DM patients compared with metformin treatment (Xie et al., in-press). However, the mechanism of PDJQ on T2DM remains unclear. In this study, we first investigated the therapeutic effect of PDJQ on T2DM rats. Then, we used 16S rRNA sequencing and untargeted metabolomics analysis to investigate the mechanism of action of PDJQ in the treatment of T2DM.

## Materials And Methods

### Reagents

High-fat high-carbohydrate (HFHC) diet (65.75% basal chow, 20% sucrose, 10% lard, 3% egg yolk powder, 1% cholesterol, 0.25% pig bile salt) was obtained from Beijing Sibeifu Bioscience Co., Ltd. (Beijing, China). Streptozotocin (STZ) and metformin were purchased from Solarbio Biotechnology Co., Ltd. (Beijing, China). Triglyceride (TG), total cholesterol (TC), low density lipoprotein (LDL), high density lipoprotein (HDL), aspartate aminotransferase (AST), alanine aminotransferase (ALT), creatinine (Cr), blood urea nitrogen (BUN), superoxide dismutase (SOD), methane dicarboxylic aldehyde (MDA), and glutathione peroxidase (GSH-Px) assay kits test kits were obtained from Nanjing Jiancheng Biological Engineering Institute (Nanjing, China). Rat insulin, interleukin (IL)-1β, IL-6, tumor necrosis factor alpha (TNF-α) enzyme-linked immunosorbent assay (ELISA) kit was obtained from Multi Science Biotechnology Co., Ltd. (Hangzhou, China). Reference standards of astragaloside, heterophyllin B, atractylodin, harpagide, berberine, baicalin, puerarin, quercetin, protocatechuic acid and tanshinone II A were obtained from Sichuan Weikeqi Biological Technology Co., Ltd. (Sichuan, China).

### Preparation of PDJQ

For the preparation of PDJQ, the following components were used:12 g of *Astragalus mongholicus* Bunge, 12 g of *Peudostellaria heterophylla* (Miq.) Pax, 6 g of *Atractylodes lancea* (Thunb.) DC., 12 g of *Scrophularia ningpoensis* Hemsl., 12 g of *Coptis chinensis* Franch., 12 g of *Scutellaria baicalensis* Georgi, 12 g of *Pueraria montana var. lobata* (Willd.) Maesen & S.M.Almeida ex Sanjappa & Predeep, 12 g of *Potentilla discolor* Bunge, 12 g of *Litchi chinensis* Sonn., and 12 g of *Salvia miltiorrhiza* Bunge. Eight volumes of water were added to this mixture, followed by decocting for 30 min and concentrating it to 6 g of crude drug/mL.

Quality control of PDJQ was performed using ultra performance liquid chromatography (UPLC; ACQUITY UPLC®, United States) coupled with Xevo G2 quadrupole time-of-flight (Q-TOF) mass spectrometer (MS; Waters Corp., Milford, United States) systems. Briefly, the test solution was injected onto an ACQUITY UPLC BEH C_18_ column (2.1mm×100mm, 1.7μm) held at 50°C. The flow rate was 0.3 mL/min and the injection volumn was 2 μL. Mobile phase A was 0.1% formic acid aqueous solution and mobile phase B was acetonitrile contained 0.1% formic acid. The mobile phase conditions were: 0 min, 5% B; 1 min, 10% B; 6 min 60% B; 6.5 min 100% B; 10 min 100% B; 10.1 min 5% B; 13 min 5% B.

A Q-TOF MS equipped with an electrospray ionization (ESI) source was used for both positive and negative ionization scan modes (m/z ranges from 50 to 1,200 Da). The scan time was 0.2 s. The capillary voltages were 3,000 V (positive mode) and 2,200 V (negative mode) respectively. The desolvation temperature was 350°C and the source temperature was 100°C. The sample cone voltage was 40 V and the extraction cone voltage was 4V. The cone gas flow was 40 L/h and the desolvation gas flow was 800 L/h (both positive and negative modes).

### Grouping and Dosing

Sixty 6–8-week-old specific pathogen free (SPF)-grade Sprague-Dawley (SD) male rats (180–220 g) were purchased from Beijing Huafukang Biotechnology Co., Ltd. (License number: SCXK 2020-0006). The feeding environment was maintained at 21±2°C with relative humidity of 45±10%. During the 12 h light-dark cycle, the rats had free access to water and food. The experiment was approved by the Ethics Committee of Tianjin University of Traditional Chinese Medicine.

**Figure 1:**
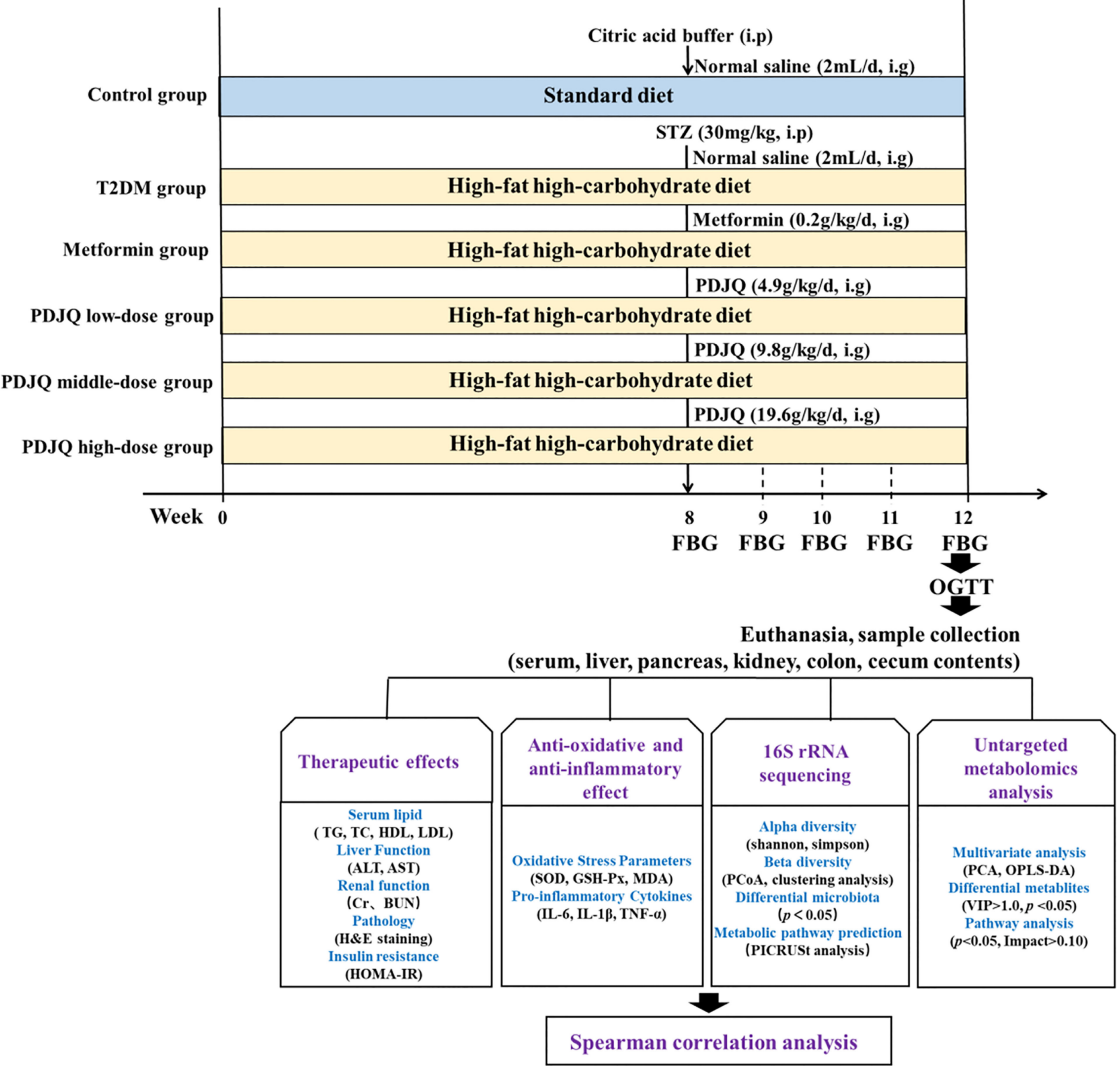
Overview of the experimental design for all groups.

After 1 week of acclimation, 10 rats were randomly selected as the control group and fed with normal chow, while the remaining 50 rats were fed with HFHC diet for 8 weeks. All rats were subsequently fasted for 12 h and given a single intraperitoneal injection of STZ at 30 mg/kg (dissolved in 0.1 mol/L citric acid buffer, pH = 4.5). The control group was injected with the same dose of citric acid buffer. Blood was collected from the tail vein 72 h after STZ injection to measure the random blood glucose level, and the model was successfully replicated with blood glucose > 16.7 mmol/L (Yang et al., 2020). After the animal model had been established, the T2DM rats were randomly divided into, T2DM, Metformin, PDJQ low-dose, PDJQ middle-dose and PDJQ high-dose groups with 10 rats in each group. After the normal clinical dose was converted based on the body surface area, a ×6 dose was used in PDJQ middle-dose group, while the low dose was half the middle dose and high dose was double the middle dose. Animals in the control and T2DM groups were gavaged with 2 mL of saline; rats in the Metformin group were gavaged with 0.2 g/kg metformin; while rats in the PDJQ low-, middle-, and high-dose groups were gavaged with 4.9 g, 9.8 g, and 19.6 g of crude drug/kg, respectively, once a day for 4 weeks. The FBG was measured in each group every week after PDJQ treatment (**Figure 1**).

### Oral Glucose Tolerance Test (OGTT)

After 4 weeks of intervention with PDJQ, all rats were fasted without water for 8 h. The blood glucose level was measured with blood glucose testing strips, which was used as the blood glucose value at 0 min. Immediately thereafter, each rat was gavaged with 50% glucose solution (2 g/kg). The blood glucose values were measured after 30, 60, 90 and 120 min of glucose solution administration. The blood glucose-time curve was plotted and the area under the curve (AUC) of OGTT was calculated.

### Serum Biochemical Indicator Tests

After 4 weeks of intervention with PDJQ, all rats were fasted for 8 h. The rats were anesthetized by the intraperitoneal injection of pentobarbital sodium (50 mg/kg). Blood was collected from the abdominal aorta using a syringe and the collected blood was centrifuged at 3000 rpm/min for 15 min to collect the serum. The serum levels of lipid-related indicators (TG, TC, HDL, LDL), liver function-related indicators (ALT, AST), kidney function-related indicators (Cr, BUN), oxidative stress-related indicators (SOD, GSH-Px, MDA) were measured using kits based on the instructions provided (Nanjing Jiancheng Biological Engineering Institute).

### ELISA

After 4 weeks of intervention with PDJQ, the serum levels of insulin, IL-6, IL-1β, and TNF-α in each group were measured using ELISA, which was performed based on the manufacturer’s instructions (Multi Science Biotechnology Co., Ltd.). Homeostatic model assessment of insulin resistance (HOMA-IR) was calculated using the following formula: HOMA-IR = [fasting blood glucose × fasting insulin (FINS)]/22.5.

### Pathology

After 4 weeks of intervention with PDJQ, the rat liver, pancreas, kidney and colon tissues were fixed with formalin solution, paraffin-embedded, and prepared into 3-μm tissue sections, which were routinely stained with hematoxylin and eosin (H&E). The histopathological changes of each were observed under a light microscope.

### 16S rRNA Sequencing

#### Extraction of Fecal Genomic DNA

Control, T2DM and PDJQ high-dose groups were selected for intestinal microbiota study. Briefly, six rats were randomly selected from each group and their cecum contents were collected and weighed. The total genomic DNA was extracted by the CTAB/SDS method, and the concentrations of DNA samples were detected using 1% agarose gel. The DNA was diluted to 1 ng/µL with sterile water based on the concentration.

#### Polymerase Chain Reaction (PCR) Amplification and Sequencing of the 16S rRNA Gene

The V3–V4 region of the 16S rRNA gene was amplified using 338F (5′-ACTCCTACGGGAGGCAGCAG-3′) and 806R (5′-GGACTACHVGGGTWTCTAAT-3′) primers. The PCR amplification system included the following: 10 ng of template DNA, 0.2 µM forward and reverse primers, and 15 µL of Phusion® High-Fidelity PCR Master Mix (New England Biolabs). The reaction conditions were as follows: pre-denaturation at 98°C for 1 min; 15 cycles of denaturation at 95°C for 10 s, annealing at 50°C for 30 s, and extension at 72°C for 30 s; and then maintenance of the reaction at 72°C for 5 min; followed by storage at 4°C. The mixed PCR products were purified using Qiagen Gel Extraction Kit (Qiagen, Germany). Then, 2% agarose gel electrophoresis was used for detection. Sequencing libraries were generated using TruSeq® DNA PCR-Free Sample Preparation Kit (Illumina, USA). The library quality was assessed using a Qubit® 2.0 Fluorometer (Thermo Scientific) and Agilent Bioanalyzer 2100 system. Finally, the libraries were sequenced to by 250 bp of paired-end sequencing on the Illumina NovaSeq platform.

#### Sequencing Data Analysis

The raw sequencing data were spliced using FLASH (v1.2.7, http://ccb.jhu.edu/software/FLASH/) (Magoč and Salzberg, 2011) and sequences were quality-controlled to obtain the final effective tags. The tags were clustered using Uparse software (Version 7.0.1001, http://drive5.com/uparse/) (Edgar, 2013) to obtain the operational taxonomic units (OTUs). The Mothur-algorithm-based SILVA reference database (Version 138, http://www.arb-silva.de/) (Quast et al., 2013) was used to annotate the OTUs with taxonomic information. Multiple sequence alignments were performed using MUSCLE software (Version 3.8.31, http://www.drive5.com/muscle/) (Edgar, 2004). OTU abundance information was normalized by the sequence number corresponding to the sample with the smallest sequence. Alpha diversity index and beta diversity analysis were subsequently performed. The Wilcoxon rank-sum test was used to test for differences between groups in diversity indices, and the Kruskal–Wallis test was used (the post hoc test was chosen as Games-Howell) combined with the multiple testing method to screen for differential microbiota, where *p* < 0.05 after false discovery rate (FDR) correction was considered to indicate a significant difference. Finally, Phylogenetic Investigation of Communities by Reconstruction of Unobserved States database (PICRUSt) analysis was applied to predict the relevant gene pathways that may be affected by each strain of differential microbiota.

### Metabolomic Analysis

#### Serum Sample Processing

Control, T2DM and PDJQ high-dose groups were selected for metabolomics study. Briefly, six rats were randomly selected from each group and their serum samples were collected. A total of 100 μL of serum sample was added to 400 μL of 80% methanol solution, vortexed and oscillated, left to stand for 5 min in an ice bath, and then centrifuged for 20 min (15,000 g, 4°C). After centrifugation, the supernatant was diluted with ultrapure water to 53 % methanol and then centrifuged at 15,000 g for 20 min at 4°C. The supernatant was collected for 20 min and used as the testing sample. Quality control (QC) sample was prepared by mixing all samples in equal amounts in order to ensure the stability and accuracy of the measurement throughout the analysis.

#### Chromatography and Mass Spectrometry Conditions

The chromatography was performed on a Hypersil Goldcolumn (C_18_) column (2.1 mm × 100 mm, 1.9 μm) with a mobile phase consisting of (A) 0.1% formic acid and (B) methanol. Gradient elution was used, as follows: 0 min, 98% A, 2% B; at 1.5 min, 98% A, 2% B; at 12 min, 0% A, 100% B; at 14 min, 0% A, 100% B; at 14.1 min, 98% A, 2% B; and at 17 min, 98% A, 2% B. The column temperature was set at 40°C, the flow rate was 0.2 mL/min, and the injection volume was 2 μL. The mass spectrometry conditions were as follows: simultaneous detection in positive and negative ion modes using an ESI source. The ESI source settings were as follows: spray voltage: 3.2 kV; sheath gasflow rate: 40 arb; aux gasflow rate: 10 arb; capillary temp: 320°C. QC samples were injected every six samples throughout the analytical run, and the data obtained were used to evaluate stability.

#### Data Processing and Analysis

Chromatographic characteristic peaks in the samples were detected using high-resolution mass spectrometry. The raw files obtained by MS were imported into Compound Discoverer 3.1 (CD3.1, Thermo Fisher) software for data pre-processing. First, the data were briefly screened by retention time and mass-to-charge ratio parameters, and then the peaks were aligned according to the retention time deviation of 0.2 min and mass deviation (parts per million, ppm) of 5 ppm for different samples to make the identification more accurate; subsequently, the data were matched according to the set values of 5 ppm, signal intensity deviation of 30%, signal-to-noise ratio (S/N) of 3, minimum signal intensity of 100,000, and adduct ions for peak extraction, and also for the quantification of peak area. From molecular ion peaks and fragment ions, the prediction of the molecular formula was performed and compared to the mzCloud (https://www.mzcloud.org/), mzVault, and MassList databases to identify the metabolites. Metabolites with a coefficient of variance of less than 30% (Dai et al., 2017) in QC samples were then retained as final identification results for subsequent analysis. The peaks detected in the samples were integrated using CD3.1, where the peak area of each characteristic peak represented the relative quantitative value of a metabolite, and the quantitative results were normalized using the total peak area, and finally the quantitative results of the metabolites were obtained. The data were then subjected to QC to ensure the accuracy and reliability of the results. Next, the metabolites were subjected to multivariate statistical analysis, including principal component analysis (PCA) and orthogonal partial least squares discriminant analysis (OPLS-DA), to reveal differences in metabolic patterns between different groups. Variable importance of projection (VIP) of each metabolites were obtained based on the OPLS-DA model. The normalized peak areas for each metabolite were also analyzed using two-tailed Student’s t-test. Differential metabolites among each group were screened based on *p* < 0.05 and, where VIP > 1. Metabolic pathway enrichment analysis was performed for differential metabolites with fold change (FC) > 1.25 or FC < 0.80 based on MetaboAnalyst software (https://www.metaboanalyst.ca/) and Kyoto Encyclopedia of Genes and Genomes (KEGG) database (https://www.kegg.jp/).

### Statistics

All data were reported as the mean ± standard deviation (mean ± SD) for the independent experiments. Statistical differences between the experimental groups were accessed by one-way analysis of variance (ANOVA) and post-hoc analysis using SPSS software (version 20.0). A *p*-value < 0.05 was considered statistically significant. Curve fitting was performed using the GraphPad Prism 5 software.

## Results

### Identification of Main Compounds in PDJQ by UPLC-MS Analysis

Astragaloside, heterophyllin B, atractylodin, harpagide, berberine, baicalin, puerarin, quercetin, protocatechuic acid and tanshinone II A were used as the reference standards to validate the main compounds in PDJQ. The detailed information of these compounds were shown in **Figure S1**. The typical based peak intensity (BPI) chromatograms of PDJQ and these reference standards were shown in **Figure S2**. The characteristic fragment ions of these compounds were shown in **Table S2** (Supplementary material). Astragaloside in *ragalus mongholicus* Bunge, heterophyllin B in *Pseudostellaria heterophylla* (Miq.) Pax, atractylodin in *Atractylodes lancea* (Thunb.) DC., harpagide in *Scrophularia ningpoensis* Hemsl., berberine in *Coptis chinensis* Franch., baicalin in *Scutellaria baicalensis* Georgi, puerarin in *Pueraria montana var. lobata* (Willd.) Maesen & S.M.Almeida ex Sanjappa & Predeep, quercetin in *Potentilla discolor* Bunge, protocatechuic acid in *Litchi chinensis* Sonn. and tanshinone II A in *Salvia miltiorrhiza* Bunge were identified as the preeminent compounds in PDJQ.

### Effects of PDJQ on Blood Glucose, Blood Lipids, Liver and Kidney Functions, IR, and Pathological Manifestations in T2DM Rats

**Figure 2:**
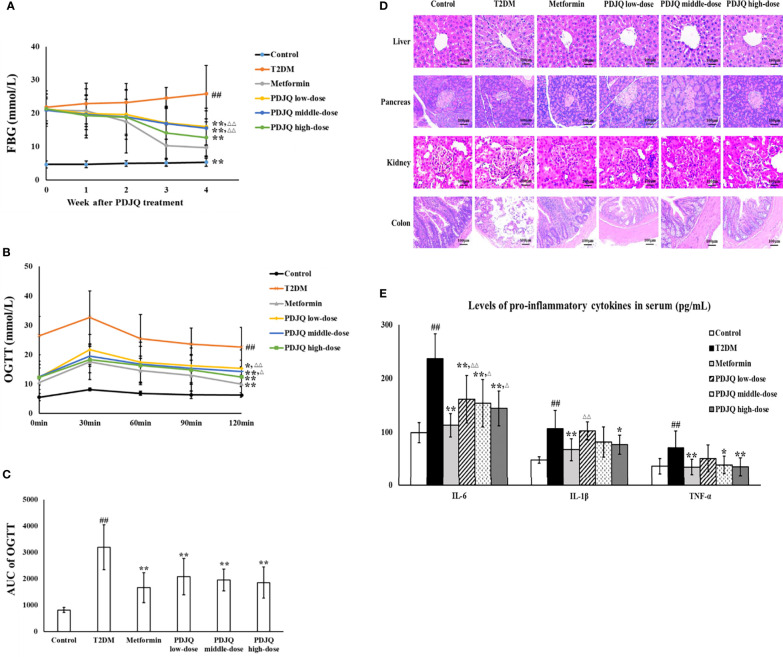
PDJQ treatment alleviated the hyperglycemia, IR, pathological changes and inflammatory response in T2DM rats. **(A)** PDJQ treatment decreased the FBG in T2DM rats. **(B, C)** The AUC of OGTT was decreased in T2DM rats after metformin and PDJQ treatment. **(D)** PDJQ treatment significantly improved the pathological changes of liver, pancreas, kidney, and colon in T2DM rats (200×). **(E)** PDJQ treatment decreased the levels of pro-inflammatory cytokines in serum. Control, T2DM, Metformin, PDJQ low-dose, PDJQ middle-dose and PDJQ high-dose (n = 10 per group) groups. Data are presented as the mean ± SD. ^##^ *p* < 0.01 as compared to the control group; ^**^ *p* < 0.01 as compared to the T2DM group; ^△^ *p* < 0.05 as compared to the Metformin group; ^△△^ *p* < 0.01 as compared to the Metformin group.

The FBG of rats was on average > 16.7 at 72 h after STZ injection. After 4 weeks of PDJQ treatment, the levels of FBG in the Metformin, PDJQ low-dose, PDJQ middle-dose, and PDJQ high-dose groups were significantly decreased compared with those of the T2DM group (*p* <0.01, respectively, **Figure 2A**).

The serum levels of TG, TC, and LDL in T2DM group were significantly increased (*p* < 0.01, respectively), and the HDL level was significantly decreased, compared with the values in the control group (*p* < 0.05). The levels of TC, TG and LDL were decreased in Metformin (*p* < 0.01 respectively), PDJQ middle-dose (*p* < 0.05, *p* < 0.05 and *p* < 0.01, respectively) and PDJQ high-dose groups (*p* < 0.01, respectively) compared with those in T2DM group. The level of HDL was increased in Metformin (*p* < 0.05), PDJQ middle-dose(*p* < 0.05) and PDJQ high-dose (*p* < 0.01) groups compared with the T2DM group (**Table 1**).

**Table 1:** Changes in blood lipid levels after PDJQ treatment.

Group	TC (mmol/L)	TG (mmol/L)	HDL (mmol/L)	LDL (mmol/L)
Control	13.0 ± 5.2	2.1 ± 0.7	8.9 ± 2.7	1.8 ± 0.5
T2DM	27.1 ± 12.4^##^	5.4 ± 2.5^##^	4.9 ± 2.3^#^	3.9 ± 1.3^##^
Metformin	13.8 ± 5.2^**^	2.7 ± 1.1^**^	7.6 ± 1.9^*^	2.2 ± 0.6^**^
PDJQ low-dose	17.7 ± 10.7	4.5 ± 1.1^△^	5.5 ± 1.4^△^	2.6 ± 1.4
PDJQ middle-dose	14.6 ± 8.5^*^	3.5 ± 1.2^*^	7.7 ± 2.0^*^	2.3 ± 0.8^**^
PDJQ high-dose	14.0 ± 3.3^**^	2.9 ± 0.9^**^	8.1 ± 1.7^**^	2.3 ± 0.9^**^

Control, T2DM, Metformin, PDJQ low-dose, PDJQ middle-dose and PDJQ high-dose (n = 10 per group) groups. Data are presented as the mean ± SD. ^#^p < 0.05 as compared to the control group; ^##^p < 0.01 as compared to the control group; ^*^p < 0.05 as compared to the T2DM group; ^**^p < 0.01 as compared to the T2DM group; ^△^p < 0.05 as compared to the Metformin group.

TG, triglyceride; TC, total cholesterol; HDL, high density lipoprotein; LDL, low density lipoprotein; T2DM, type 2 diabetes mellitus; PDJQ, Pi-Dan-Jian-Qing decoction.

In addition, the activities of serum ALT and AST were significantly increased in T2DM rats compared with rats in the Control group (*p* < 0.01, respectively), and the activities of serum ALT and AST were significantly decreased in Metformin (*p* < 0.01, respectively), PDJQ middle-dose (*p* < 0.05 and *p* < 0.01, respectively) and PDJQ high-dose groups (*p* < 0.01, respectively) compared with those in T2DM group (**Table 2**). The serum levels of Cr and BUN were higher in the T2DM group as compared to the Control group (*p* < 0.01, respectively), and the serum levels of Cr and BUN were lower in Metformin (*p* < 0.01, respectively), PDJQ low-dose (*p* < 0.05, respectively), PDJQ middle-dose (*p* < 0.01, respectively) and PDJQ high-dose groups (*p* < 0.01, respectively) compared with rats in the T2DM group (**Table 2**).

**Table 2:** Changes in serum AST, ALT activities and serum Cr, BUN levels after PDJQ treatment.

Group	AST (U/L)	ALT (U/L)	Cr (μmol/L)	BUN (mmol/L)
Control	80.7 ± 59.5	28.9 ± 6.9	34.3 ± 14.4	3.6 ± 0.6
T2DM	215.8 ± 85.1^##^	74.4 ± 34.5^##^	93.0 ± 22.0^##^	8.3 ± 2.1^##^
Metformin	87.1 ± 46.2^**^	35.4 ± 11.9^**^	53.0 ± 16.5^**^	5.0 ± 1.0^**^
PDJQ low-dose	173.7 ± 38.4^△△^	46.5 ± 23.7^*^	71.8 ± 16.0^* △^	6.0 ± 1.8^*^
PDJQ middle-dose	126.1 ± 73.2^*^	38.9 ± 15.6^**^	61.8 ± 20.0^**^	5.7 ± 1.5^**^
PDJQ high-dose	106.6 ± 43.5^**^	37.6 ± 15.8^**^	59.6 ± 12.2^**^	5.4 ± 1.7^**^

Control, T2DM, Metformin, PDJQ low-dose, PDJQ middle-dose and PDJQ high-dose (n = 10 per group) groups. Data are presented as the mean ± SD. ^##^p < 0.01 as compared to the control group; ^*^p < 0.05 as compared to the T2DM group; ^**^p < 0.01 as compared to the T2DM group; ^△^p < 0.05 as compared to the Metformin group; ^△△^p < 0.01 as compared to the Metformin group.

AST, aspartate aminotransferase; ALT, alanine aminotransferase; Cr, creatinine; BUN, blood urea nitrogen.

OGTT showed that the AUC of OGTT was significantly higher in the T2DM group than that in Control group (*p* < 0.01), and the AUC of OGTT was lower in Metformin, PDJQ low-dose, PDJQ middle-dose and PDJQ high-dose groups compared with rats in the T2DM group (*p* < 0.01, respectively, **Figures 2B, C**). In addition, compared with those in the Control group, the FINS level and HOMA-IR indicator were significantly increased in the T2DM group (*p* < 0.01, respectively). The FINS level was reduced in Metformin, PDJQ middle-dose and PDJQ high-dose groups compared with the T2DM group (*p* < 0.01, respectively, **Table 3**). The HOMA-IR was lower in Metformin, PDJQ low-dose, PDJQ middle-dose and PDJQ high-dose groups compared with the T2DM group (*p* < 0.01, respectively, **Table 3**).

**Table 3:** Levels of FINS and HOMA-IR after PDJQ treatment.

Group	FINS (μ IU/mL)	HOMA-IR
Control	6.1 ± 3.5	1.5 ± 1.0
T2DM	15.0 ± 4.6^##^	16.6 ± 6.1^##^
Metformin	7.0 ± 3.8^**^	3.0 ± 1.8^**^
PDJQ low-dose	11.7 ± 5.5	8.7 ± 5.2^** △△^
PDJQ middle-dose	8.8 ± 2.7^**^	5.8 ± 2.2^** △△^
PDJQ high-dose	7.5 ± 3.2^**^	4.2 ± 2.1^**^

Control, T2DM, Metformin, PDJQ low-dose, PDJQ middle-dose and PDJQ high-dose (n = 10 per group) groups. Data are presented as the mean ± SD. ^##^p < 0.01 as compared to the control group; ^**^p < 0.01 as compared to the T2DM group; ^△△^p < 0.01 as compared to the Metformin group.

HOMA-IR, homeostatic model assessment of insulin resistance; FINS, fasting insulin.

H&E staining showed that rats in T2DM group exhibited significant hepatocyte steatosis, hepatocyte necrosis and infiltration of inflammatory cell in liver, islet atrophy and islet cell injury in pancreas, hyperplasia of glomerular basement membrane, renal tubular atrophy, and massive inflammatory cell infiltration in kidney, damage of epithelial cells and incomplete cell arrangement in colon. The intervention of metformin and PDJQ significantly improved the histopathological changes of liver, pancreas, kidney, and colon in T2DM rats (**Figure 2D**).

### Effects of PDJQ on Oxidative Stress and Inflammatory Factors in T2DM Rats

We further evaluated the effects of PDJQ on oxidative stress in T2DM rats by measuring SOD, MDA, and GSH-Px in the serum of each group of rats. Compared with the Control group, the activities of SOD (*p* < 0.01) and GSH-Px (*p* < 0.01) in rats’ serum were significantly decreased and the level of MDA (*p* < 0.01) was significantly increased in the T2DM group. The activities of SOD and GSH-Px were elevated in Metformin (*p* < 0.01, respectively), PDJQ middle-dose (*p* < 0.05, respectively) and PDJQ high-dose (*p* < 0.01, respectively) groups compared with the T2DM group, whereas the level of MDA was reduced in Metformin (*p* < 0.01), PDJQ middle-dose (*p* < 0.05) and PDJQ high-dose (*p* < 0.01) groups compared with the T2DM group (**Table 4**). Furthermore, the levels of pro-inflammatory factors IL-6, IL-1β, and TNF-α in serum were measured to study the effects of PDJQ on the inflammatory response in T2DM rats. Compared with the control group, the levels of IL-6, IL-1β, and TNF-α in the serum of T2DM rats were significantly increased (*p* < 0.01, respectively). The level of IL-6 was lower in Metformin, PDJQ low-dose, PDJQ middle-dose and PDJQ high-dose groups compared with the T2DM group (*p* < 0.01, respectively). The level of IL-1β was lower in Metformin (*p* < 0.01) and PDJQ high-dose (*p* < 0.05) groups compared with the T2DM group. The level of TNF-α was lower in Metformin (*p* < 0.01), PDJQ middle-dose (*p* < 0.05) and PDJQ high-dose (*p* < 0.01) groups compared with the T2DM group (**Figure 2E**). The above results demonstrated that PDJQ had therapeutic effects on T2DM rats, which were most significant at high doses. Therefore, PDJQ high-dose group was selected for the follow-up intestinal microbiota and metabolomic studies. Rats selected for 16S rRNA sequencing and metabolomics analysis showed statistical significances in blood lipid levels (TG, TC, HDL, LDL, **Table S2**), liver (ALT, AST) and kidney (Cr, BUN) function-related indicators (**Table S3** **)**, FBG (**Figure S3A**), AUC of OGTT (**Figure S3B, C**), FINS and HOMA-IR (**Table S4**) between Control and T2DM groups and between T2DM and PDJQ high-dose groups.

**Table 4:** Changes in serum SOD, GSH-Px activities and serum MDA level after PDJQ treatment.

Group	SOD (U/mL)	MDA (nmol/mL)	GSH-Px (μmol/L)
Control	175.1 ± 21.0	4.3 ± 0.8	96.9 ± 12.0
T2DM	116.6 ± 31.0^##^	13.1 ± 6.1^##^	60.8 ± 16.8^##^
Metformin	166.0 ± 32.5^**^	6.2 ± 1.9^**^	90.3 ± 21.0^**^
PDJQ low-dose	132.5 ± 33.5^△^	10.2 ± 4.6^△^	70.5 ± 18.1^△^
PDJQ middle-dose	156.8 ± 47.9^*^	8.5 ± 2.5^* △^	83.2 ± 25.5^*^
PDJQ high-dose	159.8 ± 28.9^**^	6.9 ± 1.5^**^	84.2 ± 13.6^**^

Control, T2DM, Metformin, PDJQ low-dose, PDJQ middle-dose and PDJQ high-dose (n = 10 per group) groups. Data are presented as the mean ± SD. ^##^p < 0.01 as compared to the control group; ^*^p < 0.05 as compared to the T2DM group; ^**^p < 0.01 as compared to the T2DM group; ^△^p < 0.05 as compared to the Metformin group.

SOD, superoxide dismutase; MDA, methane dicarboxylic aldehyde; GSH-Px, glutathione peroxidase.

### Effects of PDJQ on the Intestinal Microbiota of T2DM Rats

**Figure 3:**
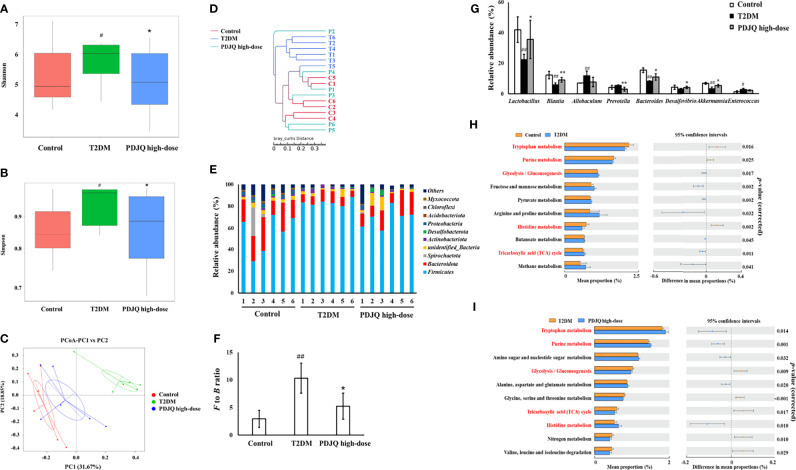
PDJQ treatment affected the gut microbiota community in T2DM rats. **(A, B)** Shannon and simpson indexes were lower in PDJQ high-dose group than that in the T2DM group. **(C, D)** PCoA and system clustering tree indicated more similar beta diversity between PDJQ high-dose and Control groups than that between the T2DM and Control groups (C: Control group; M: T2DM group; P: PDJQ high-dose group). **(E, F)** At the phylum level, PDJQ treatment decreased the *F* to *B* ratio in T2DM rats. **(G)** At the genus level, PDJQ treatment affected the relative abundances of *Lactobacillus*, *Blautia, Prevotella, Bacteroides, Desulfovibrio* and *Akkermansia* in T2DM rats. **(H, I)** The differential metabolic pathways (written in red) of PDJQ on T2DM were predicted using PICRUSt analysis based on the 16S rRNA sequencing data. Control, T2DM and PDJQ high-dose (n = 6 per group) groups. Data are presented as the mean ± SD. ^#^ *p* < 0.05 as compared to the control group; ^##^ *p* < 0.01 as compared to the control group; ^*^ *p* < 0.05 as compared to the T2DM group; ^**^ *p* < 0.01 as compared to the T2DM group.

16S rRNA sequencing was used to investigate the changes of intestinal microbiota in T2DM rats after PDJQ treatment. The results showed that the Shannon and Simpson indexes were significantly higher in the T2DM group than in the Control group (*p* < 0.05) and significantly lower in the PDJQ high-dose group than in the T2DM group (*p* < 0.05, **Figures 3A, B**). The principal co-ordinates analysis (PCoA) results showed that sample points of the T2DM group could be completely separated from the Control group, and the sample points of the PDJQ high-dose group were very close to those of the Control group. Likewise, clustering analysis also demonstrated that the distance from the Control group to the PDJQ high-dose group was shorter than the distance from the Control group to the T2DM group (**Figures 3C, D**). The above results indicated that the overall structure and composition of the intestinal microbiota of T2DM rats had changed significantly, and the high dose of PDJQ could effectively reverse this change.

The composition of the intestinal microbiota in each group of samples at the phylum level is shown in the **Figure 3E**, showing *Firmicutes* and *Bacteroidetes* as the dominant ones. The *Firmicutes/Bacteroidetes* (*F* to *B*) ratio was significantly higher in the T2DM group than in the Control group (*p* < 0.01), while it was significantly lower in the PDJQ high-dose group than in the T2DM group (p < 0.05, **Figure 3F**). At the genus level, the abundances of *Allobaculum* (*p* < 0.01) and *Enterococcus* (*p* < 0.05) were higher, while those of *Lactobacillus* (*p* < 0.01), *Blautia* (*p* < 0.01), *Bacteroides* (*p* < 0.01), and *Akkermansia* (*p* < 0.01) were lower in the T2DM group than in the Control group. Compared with the T2DM group, the abundances of *Lactobacillus* (*p* < 0.05), *Blautia* (*p* < 0.01), *Bacteroides* (*p* < 0.05), *Desulfovibrio* (*p* < 0.05), and *Akkermansia* (*p* < 0.05)were increased, while that of *Prevotella* (*p* < 0.01) was decreased in PDJQ high-dose group (**Figure 3G**).

PICRUSt analysis was used to predict the functional changes of intestinal microbiota in T2DM rats after the intervention with a PDJQ. The results of the prediction based on the KEGG database showed that a total of six categories of biological metabolic pathway functional analyses were obtained at the first functional layer, which were Cellular Processes, Environmental Information Processing, Genetic Information Processing, Human Diseases, Metabolism, and Organismal Systems. The top ten metabolic pathways with the highest proportions and *p*-values < 0.05 are listed in the **Figure 3H** (Control group *vs.* T2DM group) and **Figure 3I** (T2DM group *vs.* PDJQ high-dose group). Proportions of metabolic pathways that were increased in the T2DM group compared with Control group but decreased in PDJQ high-dose group compared with T2DM group, or vice versa, were considered as differential pathways. The abundances of glycolysis/gluconeogenesis and tricarboxylic acid (TCA) cycle pathways were all increased in the T2DM group compared with the Control group. The abundances of purine metabolism, tryptophan metabolism and histidine metabolism pathways were lower in T2DM group compared with the Control group (**Figure 3H**). For the PDJQ high-dose group, the abundances of purine metabolism, trptophan metabolism and histidine metabolism pathways were increased and the abundances of glycolysis/gluconeogenesis and TCA cycle pathways were decreased compared with the T2DM group (**Figure 3I**).

### Effects of PDJQ on the Levels of Serum Metabolites in T2DM Rats

**Figure 4:**
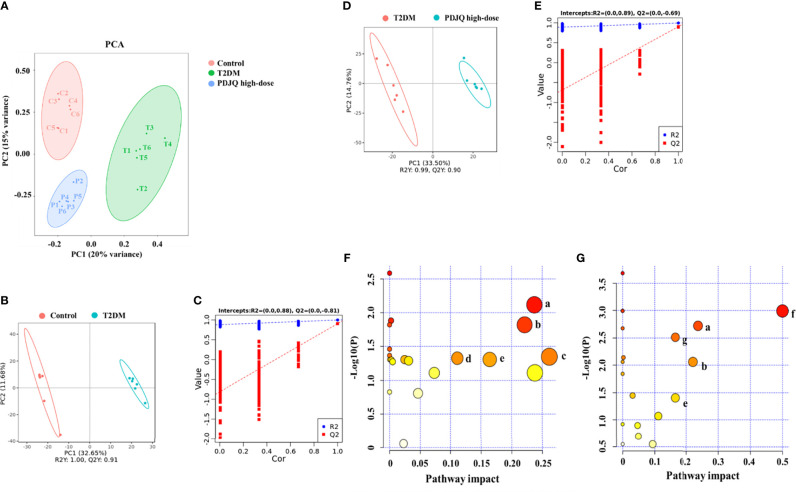
PDJQ treatment regulated the serum metabolites in T2DM rats. **(A)** Scores plots of PCA between the control and T2DM groups and the T2DM and PDJQ high-dose groups. **(B, C)** Scores plots of OPLS−DA between the Control and T2DM groups and the corresponding coefficient of loading plots. **(D, E)** Scores plots of OPLS−DA between the T2DM and PDJQ high-dose groups and the corresponding coefficient of loading plots. **(F, G)** Summary of pathway analysis of serum samples between control and T2DM groups and between T2DM and PDJQ high-dose groups. **(A)**Tryptophan metabolism; **(B)** Histidine metabolism; **(C)** Glycerolipid metabolism; **(D)** Glyoxylate and dicarboxylate metabolism; **(E)** TCA cycle; **(F)** Phenylalanine, tyrosine and tryptophan biosynthesis; **(G)** Tyrosine metabolism. Control, T2DM and PDJQ high-dose (n = 6 per group) groups.

PCA generates new characteristic variables using linear combinations of metabolite variables with certain weights, and groups of data are categorized by major new variables (principal components). The PCA model can reflect the original state of metabolomic data, and the degree of aggregation and dispersion of the samples can be observed from the PCA model. The PCA plot showed that the Control group could be well distinguished from the T2DM group, and the T2DM group and the PDJQ high-dose group were also well distinguished (**Figure 4A**). To identify the differential metabolites, the OPLS-DA model was used. In agreement with the PCA results, OPLS-DA models showed significant distinctions of metabolomic data between the Control and the T2DM group as well as between the T2DM group and the PDJQ high-dose group (**Figures 4B, D**). In addition, the explanatory rate (R^2^) and predictive power (Q^2^) of OPLA-DA model were evaluated using seven-round cross validation and 200 repetitions of response permutation testing (RPT). Compared with the Control group, the T2DM group had R^2^ = 0.88 and Q^2^ = −0.81 **(** **Figure 4C** **)**. Compared with the T2DM group, the PDJQ high-dose group had R^2^ = 0.89 and Q^2^ = −0.69 (**Figure 4E**). These results indicated that the OPLS-DA model was stable and had good predictive ability.

The detailed trends of the changes of differential metabolites (FC > 1.2 or FC < 0.8, *p* < 0.05 and VIP > 1.0) are shown in **Table 5**. We also used MetaboAnalyst software to screen the differential metabolic pathways to screen for significant metabolic pathways (*p* < 0.05, impact value > 0.10) of the differential metabolites. Compared with the control group, the metabolic pathways that were altered in the T2DM group mainly included the metabolism of tryptophan, histidine, glycerolipid, glyoxylate, and dicarboxylate and the TCA cycle (**Figure 4F**); while the metabolic pathways affected by PDJQ mainly included tryptophan metabolism, histidine metabolism, the TCA cycle, phenylalanine, tyrosine, and tryptophan biosynthesis, and tyrosine metabolism (**Figure 4G**). The same metabolic pathway of PICRUSt analysis that had done 16S rRNA sequencing and untargeted metabolomics pathway analysis included tryptophan metabolism, histidine metabolism, and the TCA cycle and were discussed in detail.

**Table 5:** The differential metabolites in serum after PDJQ treatment.

No.	Rt (min)	m/z	Formula	Metabolites	VIP		FC		Trend		Pathway
					T *vs*. C	P *vs*. T	T *vs*. C	P *vs*. T	T *vs*. C	P *vs*. T	
**1**	1.7	145.1	C_6_H_14_N_2_O_2_	L-lysine	1.3	1.6	0.5	2.2	↓#	↑**	–
**2**	1.8	228.1	C_9_H_13_N_3_O_4_	Deoxycytidine	1.8	0.9	0.4	1.5	↓##	↑*	–
**3**	1.4	146.1	C_7_H_15_NO_2_	Acetylcholine	1.7	1.6	0.5	1.8	↓##	↑**	–
**4**	1.1	133.1	C_5_H_12_N_2_O_2_	Ornithine	1.5	0.8	0.3	1.8	↓##	↑	–
**5**	1.2	131.1	C_4_H_8_N_2_O_3_	Asparagine	1.9	1.6	0.5	1.7	↓##	↑**	–
**6**	1.3	125.0	C_3_H_6_O_3_	D-Glyceraldehyde	1.4	1.7	0.4	0.9	↓##	↓	c
**7**	11.1	464.3	C_26_H_43_NO_6_	Glycocholic acid	1.3	0.3	3.7	0.7	↑#	↓	–
**8**	5.9	203.1	C_11_H_12_N_2_O_2_	DL-Tryptophan	1.7	1.3	0.4	1.9	↓##	↑*	a
**9**	8.4	209.1	C_10_H_12_N_2_O_3_	L-Kynurenine	1.6	2.2	0.1	13.8	↓#	↑**	a
**10**	1.2	133.0	C_4_H_6_O_5_	D-(+)-Malic acid	1.3	1.7	1.8	0.5	↑##	↓**	d, e
**11**	6.0	191.0	C_6_H_8_O_7_	Citric acid	1.5	1.1	1.7	0.7	↑##	↓*	d, e
**12**	1.2	115.0	C_4_H_4_O_4_	Fumaric acid	1.3	1.6	3.1	0.3	↑##	↓**	e, g
**13**	1.3	120.1	C_4_H_9_NO_3_	L-Threonine	1.2	1.2	0.4	1.7	↓##	↑*	–
**14**	1.4	132.1	C_4_H_9_N_3_O_2_	Creatine	1.4	1.7	1.6	1.1	↑#	↑	–
**15**	6.0	122.1	C_8_H_11_N	2-Phenylethylamine	2.5	0.6	0.3	1.3	↓##	↑**	–
**16**	8.1	194.1	C_10_H_11_NO_3_	Phenylacetylglycine	2.1	0.9	5.9	0.5	↑##	↓*	–
**17**	1.5	116.1	C_5_H_9_NO_2_	D-Proline	1.3	0.8	0.6	1.2	↓#	↑	–
**18**	1.4	147.1	C_5_H_10_N_2_O_3_	D-(-)-Glutamine	1.3	2.0	0.7	1.6	↓##	↑**	–
**19**	4.1	153.0	C_5_H_4_N_4_O_2_	Xanthine	0.6	1.6	2.0	0.1	↑	↓**	–
**20**	6.6	221.1	C_11_H_12_N_2_O_3_	5-Hydroxytryptophan	1.2	1.5	0.6	1.6	↓#	↑**	–
**21**	1.3	105.0	C_3_H_6_O_4_	L-(-)-Glyceric acid	0.6	1.1	1.2	1.6	↑	↑**	–
**22**	8.9	216.1	C_6_H_14_ClNO_5_	D-Galactosamine	0.4	1.1	0.5	2.5	↓	↑**	–
**23**	1.3	167.0	C_5_H_4_N_4_O_3_	Uric acid	0.2	1.3	1.2	0.5	↑	↓*	–
**24**	1.4	132.0	C_5_H_9_NO_3_	Hydroxyproline	1.2	1.7	1.4	1.1	↑#	↑	–
**25**	2.0	180.1	C_9_H_11_NO_3_	L-Tyrosine	1.0	1.3	0.5	1.7	↓#	↑**	f, g
**26**	14.5	542.3	C_28_H_50_NO_7_P	Lysopc 20:4	1.9	1.6	0.3	2.3	↓#	↑**	–
**27**	1.3	105.0	C_3_H_6_O_4_	L-(-)-Glyceric acid	1.6	1.1	1.9	1.6	↑##	↑	c, d
**28**	1.7	154.1	C_6_H_9_N_3_O_2_	L-Histidine	1.6	1.5	0.5	1.9	↓##	↑*	b

Control, T2DM and PDJQ high-dose (n = 6 per group) groups.

^#^p < 0.05 as compared to the control group; ^##^p < 0.01 as compared to the control group; ^*^p < 0.05 as compared to the T2DM group; ^**^p < 0.01 as compared to the T2DM group; ↑, content increased; ↓, content decreased; *vs*, *versus*; C, control group; T, T2DM group; P, PDJQ high-dose group; Rt, retention time; VIP, variable importance of projection; FC, fold change.

**a:** Tryptophan metabolism; **b:** Histidine metabolism; **c:** Glycerolipid metabolism; **d:** Glyoxylate and dicarboxylate metabolism; **e:** TCA cycle; **f**: Phenylalanine, tyrosine and tryptophan biosynthesis; **g**: Tyrosine metabolism.

### Correlation Analysis of Physiological Data, Oxidative Stress and Inflammatory Factors, Untargeted Metabolomics and Gut Microbiota

**Figure 5:**
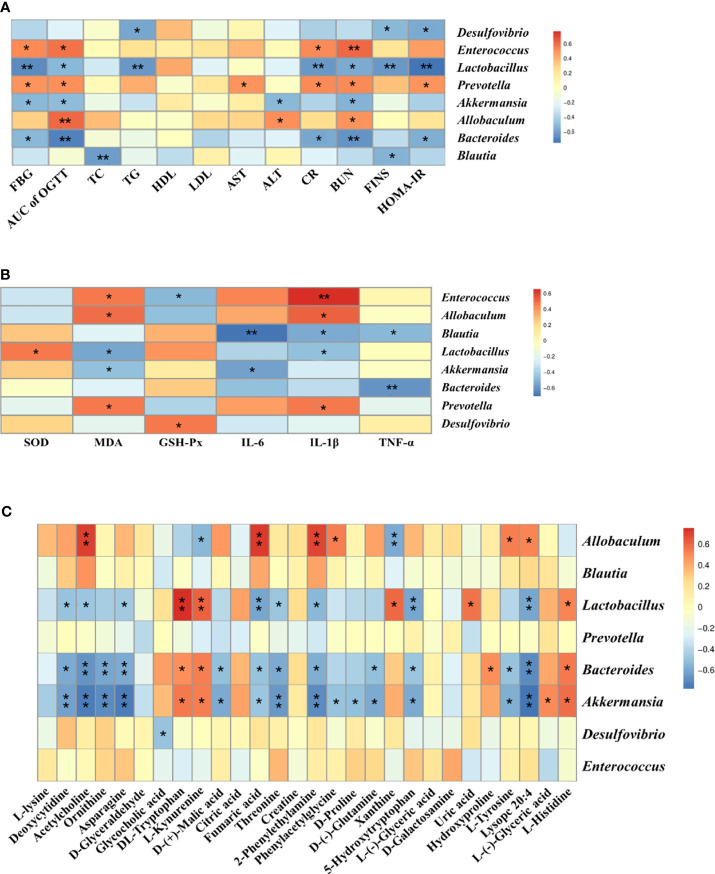
Correlation analysis of physiological data, oxidative stress and inflammatory factors, untargeted metabolomics and 16S rRNA sequencing. Correlations between physiological data and gut microbiota **(A)**, between oxidative stress and inflammatory factors and gut microbiota **(B)** and between untargeted metabolomics and gut microbiota **(C)** were analyzed using spearman’s analysis (heatmap). X-axis represents the physiological indices **(A)**, oxidative stress and inflammatory factors **(B)** and differential metabolites in the serum **(C)**. Y-axis represents the gut microbiota with differential abundance. The colors of grids represent the correlation analysis value of spearman’s correlation analysis. Grids in red indicate positive correlations (correlation analysis value more than 0.1), while grids in blue indicate negative correlations (correlation analysis value less than -0.1). Color coding scale indicates the correlation analysis value from heatmap, the deeper red or blue indicates higher correlation values. *p < 0.05 between physiological data, oxidative stress and inflammatory factors, differential serum metabolites and gut microbiota. **p < 0.01 between physiological data, oxidative stress and inflammatory factors, differential serum metabolites and gut microbiota.

Spearman’s correlation analysis was conducted to analyze the relationship between physiological data, oxidative stress and inflammatory factors, differential serum metabolites and gut microbiota at genus level in the Control, T2DM and PDJQ high-dose groups. As shown in **Figure 5A**, *Lactobacillus*, *Bacteroides* and *Akkermansia* showed negative correlations and *Allobaculum*, *Prevotella* and *Enterococcus* showed positive correlations with most of the physiological indices. In addition, *Blautia*, *Bacteroides* and *Akkermansia* showed negative correlations and *Prevotella*, *Allobaculum* and *Enterococcus* showed positive correlations with some of the pro-inflamatory cytokines. *Desulfovibrio, Prevotella Akkermansia, Lactobacillus*, *Allobaculum* and *Enterococcus* showed correlations with some of the oxidative stress factors (**Figure 5B**)*. Allobaculum, Lactobacillus*, *Bacteroides* and *Akkermansia* showed correlations with most of the metabolites (**Figure 5C**).

## Discussion

Our results showed that, compared with the control group, the T2DM rats exhibited a significant increase in blood glucose, disorders in lipid levels and elevated ALT, AST, Cr and BUN. In addition, the HOMA-IR and AUC of OGTT were elevated in the T2DM rats, suggesting IR in rats from the T2DM group. The pathological results showed that the hepatocytes in the liver of the T2DM group rats were disordered, enlarged, and had deformation of vacuolated balloon; the pancreatic islets in the pancreas were sparsely distributed and vacuolated; the smooth muscle layer of the colon was thinned and the number of myocytes was reduced; the glomeruli in the kidney tissue were swollen and enlarged; the epithelial cells of the renal tubules were swollen or detached; the renal interstitium was infiltrated with inflammatory cells; and mesangial proliferation and interstitial fibrosis were also observed. The above results are consistent with the pathological manifestations of T2DM (Huang et al., 2014; Fan et al., 2018). The intervention of PDJQ can reduce blood glucose and improve dyslipidemia, IR, and pathological changes of kidney, liver, pancreas, and colon tissues in T2DM rats. This suggests that PDJQ has a therapeutic effect on T2DM, with the most significant findings being found in the high-dose group. In addition, metformin was selected as a positive control for T2DM in our study. Metformin is the first-line regimen for the treatment of T2DM in clinical practice (Inzucchi et al., 2012). Our results showed that there were no significant differences in FBG, FINS, HOMA-IR, and serum levels of biochemical indicators between PDJQ high-dose and Metformin groups. The small differences between the two experimental groups suggested that PDJQ has the potential to substitute for metformin in the treatment of T2DM.

Our results also showed that PDJQ could increase SOD and GSH-Px activities and decrease MDA content in T2DM rats, suggesting that this decoction helped reduce the oxidative stress response in T2DM rats. Oxidative stress is closely related to the development of T2DM (Said et al., 2020) and the development of T2DM is characterized by disorders of glucolipid metabolism, hyperglycemia, and hyperlipidemia, which lead to the production of large amounts of reactive oxygen species (ROS) in mitochondria; these in turn damage mitochondrial functions and cause oxidative stress responses. This then leads to IR by blocking insulin action pathways, and leading to the development of T2DM (Yaribeygi et al., 2020). Oxidative stress also leads to the massive production of free radicals, causing lipid peroxidation, which in turn causes cellular damage (Chen et al., 2013). MDA is a product of lipid peroxidation caused by free radicals or ROS in cells under oxidative stress, and MDA levels can indirectly reflect the degree of oxidative damage in cells (Dai et al., 2011). SOD and GSH-Px are antioxidant enzymes that reflect the strength of antioxidant capacity (Gao et al., 2018). SOD, as an intracellular oxygen radical scavenger, catalyzes the conversion of O_2_^−^ to O_2_ and H_2_O_2_, and protects the organism from superoxide anions (Ding et al., 2019). GSH-Px is an important peroxide-degrading enzyme that is widespread in the body. It catalyzes the conversion of reduced glutathione to oxidized glutathione and protects cells from peroxide disruption and damage (Tsai et al., 2019).

The results of ELISA showed that PDJQ reduced the levels of the pro-inflammatory factors IL-6, IL-1β, and TNF-α levels in sera and that the inflammatory reaction is a key factor behind the development of T2DM. The production of local and systemic pro-inflammatory cytokines such as IL-6, IL-1β, and TNF-α exacerbates the vicious cycle of T2DM by inducing IR (Baek et al., 2020). IL-6 decreases insulin activity by blocking the expression of insulin receptor signaling, leading to the development of IR (Senn et al., 2002). IL-1β contributes to increase the secretion of intercellular adhesion molecule-1 (Yang et al., 2010), which is responsible for islet beta-cell injury and death, further exacerbating diabetes. TNF-α can induce the phosphorylation of insulin receptor substrate-1 (Rotter et al., 2003), thus disrupt the metabolism of blood glucose.

16S rRNA sequencing was used to study the effects of PDJQ on the composition of intestinal microbiota in T2DM rats. The alpha diversity of intestinal microbiota refers to the diversity of flora in a specific region or ecosystem, and is a comprehensive indicator reflecting the richness and homogeneity of flora. Shannon and Simpson indexes were significantly increased in T2DM model rats, indicating that the alpha diversity of gut microbiota was increased in T2DM model rats. PDJQ treatment decreased the alpha diversity of gut microbiota. The beta diversity of intestinal microbiota of rats was analyzed by PCoA and cluster analysis, and the overall composition of intestinal microbiota in T2DM rats changed significantly. The intestinal microbiota in T2DM rats was restored to the normal level after the intervention of PDJQ. Our results are consistent with previous reports demonstrating that T2DM could alter the alpha and beta diversities of intestinal microbiota (Chen et al., 2018).

Moreover, PDJQ helps to reduce the high *F* to *B* ratio caused by T2DM. This ratio is closely linked to many diseases and significantly increased in many models, for instance, T2DM, non-alcoholic fatty liver disease, and depression (Cheung et al., 2019; Jasirwan et al., 2021; Xia et al., 2021). Metabolic disorder and inflammatory reaction can be alleviated by lowering the ratio of *F* to *B* ratio (Stojanov et al., 2020). The relative abundances of *Allobaculum* and *Enterococcus* were significantly increased, while those of *Lactobacillus*, *Blautia*, *Bacteroides*, and *Akkermansia* were significantly decreased in the T2DM rat model; PDJQ significantly increased the relative abundances of *Lactobacillus*, *Blautia*, *Bacteroides*, and *Desulfovibrio*, and *Akkermansia* and decreased the abundance of *Prevotella*. *Lactobacillus* is a probiotic that improves intestinal ecological dysregulation and oxidative stress in T2DM rats (Morshedi et al., 2020). *Blautia* is associated with anti-inflammatory effects along with many diseases including colorectal carcinoma, hepatic encephalopathy, and graft versus host disease (Jenq et al., 2015). It was found that a decrease in the intestinal probiotic *Bacteroides* was associated with abnormal glucose tolerance, and had an impact on the sugar and lipid metabolism in the body, which in turn contributed to the development of obesity and the onset of diabetes mellitus (Qiao et al., 2020). Further studies have shown that these processes are mainly related to the production of SCFAs, which are produced by intestinal microbiota breaking down oligosaccharides, polysaccharides, peptides, proteins, and glycoproteins; these in turn directly regulate the number and function of pancreatic beta-cells, and thus participate in hepatic glycogen metabolism (Liu et al., 2020). The increased abundance of *Akkermansia* reduces the elevated lipopolysaccharide levels caused by a high-fat diet and is important for maintaining glucose homeostasis (Shin et al., 2014). It has been found that metformin treatment could significantly increase the proportion of *Akkermansia* in obese mice (Lee and Ko, 2014). Likewise, our correlation analysis also showed negative correlations of *Lactobacillus*, *Blautia*, *Bacteroides*, *Desulfovibrio* and *Akkermansia* with some of the physiological indicies (FBG, IR indicies, blood lipid levels and blood biochemical indicators), oxidative stress factor (MDA) and pro-inflammatory cytokines(IL-1β, IL-6, TNF-α). Positive correlations were also found between some of the anti-oxidative factors (SOD, GSH-Px) and *Lactobacillus*, *Desulfovibrio*. Finally, *Prevotella*, a pathogenic bacterium specialized in surviving under anaerobic conditions, is usually found in oral infections and is capable of causing wound infection and purulence (Yanagisawa et al., 2006). Correlation analysis also showed positive correlations of *Prevotella* with physiological indicies (FBG, AUC of OGTT, AST, Cr, BUN, HOMA-IR), pro-inflammatory cytokine (IL-1β) and oxidative stress factor (MDA). However, the role of *Prevotella* during T2DM progression still need to be further studied. Further studies should also be carried out using fecal transplantation or gut microbiota depletion models to verify whether PDJQ can ameliorate hyperglycemia, hyperlipidemia, IR, oxidative stress and inflammatory responses to treat T2DM through regulating these gut microbiota.

The results of serum untargeted metabolomics showed that PDJQ could affect tryptophan metabolism, histidine metabolism, TCA cycle, phenylalanine, tyrosine and tryptophan biosynthesis, and tyrosine metabolism. After correlating the differential metabolic pathways obtained from metabolomics with those deduced from 16S rRNA sequencing, the pathways of tryptophan metabolism, histidine metabolism, and the TCA cycle were identified as the shared pathways, suggesting that PDJQ may affect tryptophan metabolism, histamine metabolism, and the TCA cycle through the regulation of intestinal microbiota to treat T2DM.

### Tryptophan Metabolism

Tryptophan and its related metabolites are closely associated with T2DM and IR (Chen et al., 2016). In our study, we found that the levels of DL-tryptophan and L-kynurenine were reduced in T2DM model rats and significantly increased after treatment with PDJQ. The DL-tryptophan metabolite quinolinic acid inhibits the activity of phosphoenolpyruvate carboxykinase (PEPC), which in turn inhibits gluconeogenesis and lowers blood glucose levels (Dayer et al., 2009). The majority of tryptophan in the body is metabolized by the kynurenine pathway, which is involved in inflammatory and immune reactions (Zhou and Ye, 2018). The key enzymes for the conversion of tryptophan to kynurenine are tryptophan 2,3-dioxygenase (TDO) and indoleamine 2,3-dioxygenase (IDO), which are activated by stress hormones or inflammatory factors and then slowly overproduce downstream metabolites including kynurenine. This ultimately causes the onset of diabetes (Kartika et al., 2020). The ratio of kynurenine to tryptophan is an indicator of IDO activation. When the ratio of tryptophan to kynurenine is high, the organism is more prone to inflammatory reactions and autoimmune disease (Brandacher et al., 2006). Spearman’s analysis showed a positive correlation of *Lactobacillus*, *Bacteroides* and *Akkermansia* with DL-tryptophan and L-kynurenine levels. Additionally, we observed a negative correlation between *Allobaculum* and L-kynurenine. As 16S r RNA sequencing showed that there were no significant difference in the relative abundance of *Allobaculum* between T2DM and PDJQ high-dose groups, it’s likely the modulatory effects of PDJQ on tryptophan metabolism may occur through affecting the abundances of *Lactobacillus*, *Bacteroides* and *Akkermansia*.

### Histamine Metabolism

Histidine and its metabolites are closely related to the resistance to oxidative stress and inflammatory reactions in the body (Yu et al., 2015). Our results showed that the level of L-histidine was decreased in T2DM rats and increased after the treatment with PDJQ. Histidine can inhibit TNF-α signaling pathway and attenuate the expression of pro-inflammatory cytokines (Lee et al., 2005), and it has been used to treat inflammation in obese patients with metabolic syndrome (Feng et al., 2013). In addition, histidine shows strong binding affinity for Fe^2+^ ions, thus reducing the amount of ROS produced by the Fenton reaction and protecting cells from damage caused by iron overload (Vera-Aviles et al., 2018; Wake et al., 2020). Histidine enhances the expression and activity of catalase and GSH-Px, thus reducing oxidative stress (Liu et al., 2008; Watanabe et al., 2008). Spearman’s analysis showed a positive correlation of *Lactobacillus*, *Bacteroides* and *Akkermansia* with L-histidine level. Thus, we assumed that the effects of PDJQ on L-histidine level may be associated with modulating the abundances of *Lactobacillus*, *Bacteroides* and *Akkermansia*.

### TCA Cycle

The TCA cycle is the final metabolic pathway for carbohydrates, lipids, and amino acids (Chen et al., 2014). The TCA cycle can affect the immunity of the body (Choi et al., 2012). and is closely associated with many different metabolic diseases including T2DM. In our study, we found that D-(+)-malic acid, citric acid, and fumaric acid were significantly elevated in the T2DM group of rats and decreased after administration of the medicine. Citric acid is an important intermediate in the TCA cycle, which has been found to be an important substrate for cellular energy metabolism and is closely associated with T2DM. It is a major source of adenosine triphosphate (ATP) produced by cells and has key roles in inflammation, insulin secretion, and metabolic regulation (Iacobazzi and Infantino, 2014). Citric acid can be cleaved by ATP-citrate synthase (ACLY) to acetyl coenzyme A and oxaloacetate (OAA) (Wang et al., 2021). Acetyl coenzyme A provides a basis for the synthesis of lipids, including arachidonic acid, which produces inflammatory mediators such as prostaglandins, leukotrienes, and thromboxanes (Douzi et al., 2020). OAA is the second product of citric acid and can beconverted to malic acid, which is in turn oxidized by malic enzyme to pyruvic acid to form nicotinamide adenine dinucleotide phosphate (NADPH), which can produce ROS by the action of NADPH oxidase, and the excess ROS can lead to oxidative stress (Munhoz et al., 2016). Metabolic disorder of fumaric acid may be associated with diabetic renal impairment. Fumaric acid is a dicarboxylic acid that is a precursor of L-malate during the TCA cycle. The accumulation of fumaric acid has been shown to lead to oxidative stress (Zheng et al., 2015), and prolonged oxidative stress leads to renal impairment in diabetic nephropathy. Negative correlations between *Bacteroides*, *Akkermansia* and D-(+)-malic acid levels and between *Bacteroides*, *Akkermansia Lactobacillus* and fumaric acid were found by our spearman’s analysis. *Allobaculum* showed positive correlations with fumaric acid level. The effect of PDJQ on TCA cycle in T2DM rats could be through affecting the abundances of *Bacteroides*, *Akkermansia Lactobacillus.*

## Conclusion

In conclusion, our study revealed the various ameliorative effects of PDJQ on T2DM, including improving the liver and kidney functions and alleviating the hyperglycemia, hyperlipidemia, IR, pathological changes, oxidative stress and inflammatory responses. The mechanisms of PDJQ on T2DM are likely linked to an improvement in the dysbiosis of gut microbiota and modulation of tryptophan metabolism, histamine metabolism, and the TCA cycle.

## Data Availability Statement

The datasets presented in this study can be found in online repositories. The names of the repository/repositories and accession number(s) can be found below: https://www.ncbi.nlm.nih.gov/bioproject/PRJEB48123.

## Ethics Statement

The animal study was reviewed and approved by Yunnan Hospital of Traditional Chinese Medicine Ethics Committee.

## Author Contributions

XX wrote the manuscript. XX, JL, YA, JG, and JZ conducted animal experiments. XX, JL, FQ, and CX finished molecular bioassays. ZZ, WW, HC, and HW provided technical guidance for the whole work. All authors contributed to the article and approved the submitted version.

## Funding

This work was supported by National Science Foundation of China (No. 82104802); Science and Technology Projects of Yunnan Province (No. 202101AT070246); Key scientific project of Yunnan province (No. 2019ZF005).

## Conflict of Interest

The authors declare that the research was conducted in the absence of any commercial or financial relationships that could be construed as a potential conflict of interest.

## Publisher’s Note

All claims expressed in this article are solely those of the authors and do not necessarily represent those of their affiliated organizations, or those of the publisher, the editors and the reviewers. Any product that may be evaluated in this article, or claim that may be made by its manufacturer, is not guaranteed or endorsed by the publisher.
