# Supplementary material for: Pi-Dan-Jian-Qing Decoction Ameliorates Type 2 Diabetes Mellitus Through Regulating the Gut Microbiota and Serum Metabolism
Source: Front Cell Infect Microbiol. 2021 Dec 6;11:748872. doi: 10.3389/fcimb.2021.748872 (PMC8685325; doi:10.3389/fcimb.2021.748872)
Supplement: Supplementary file 1 [file DataSheet_1.docx]

**
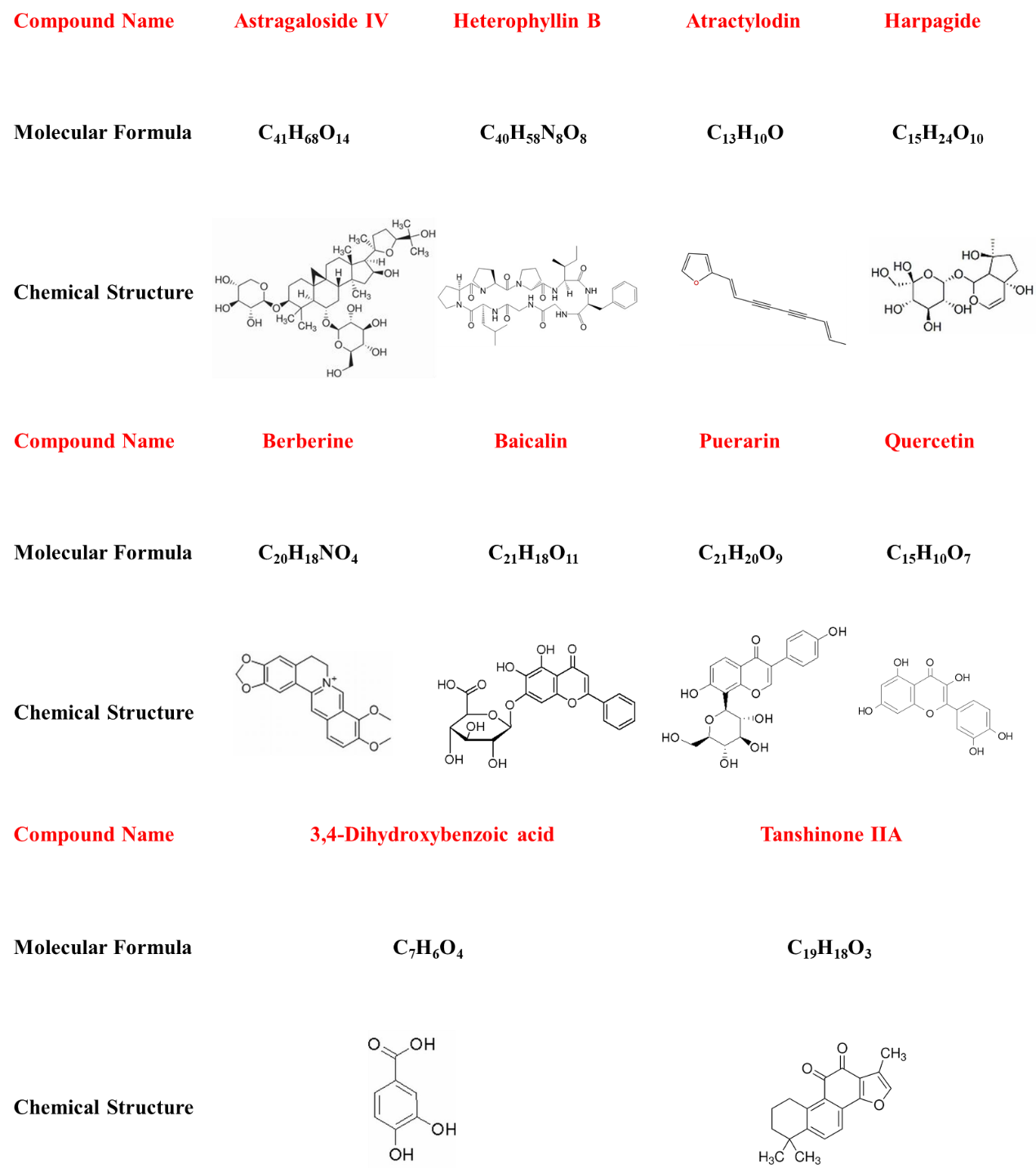
**

**FIGURE S1:** The molecular formulas and chemical structures of reference standards.

**a**

**
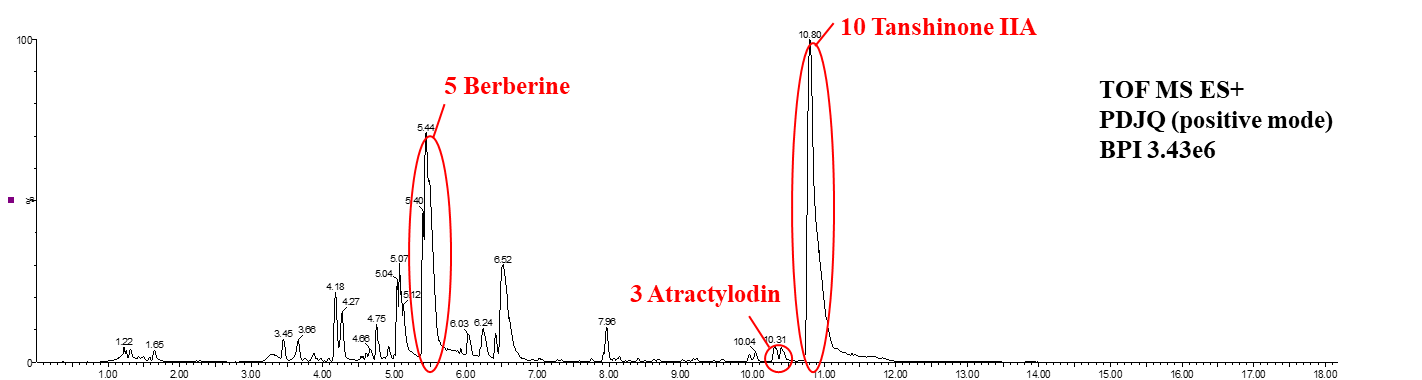
**

**b**

**
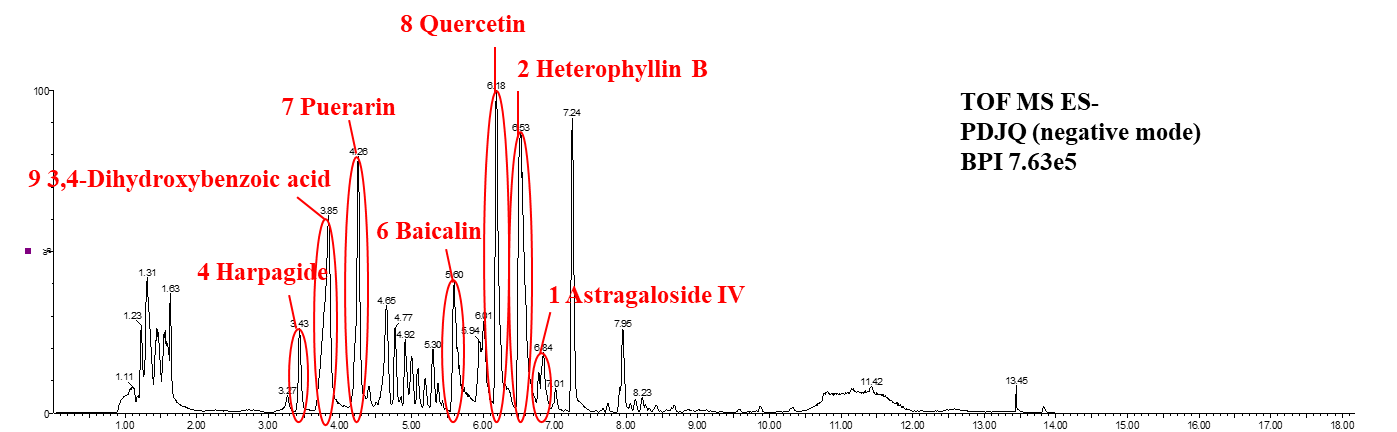
**

**c**

**
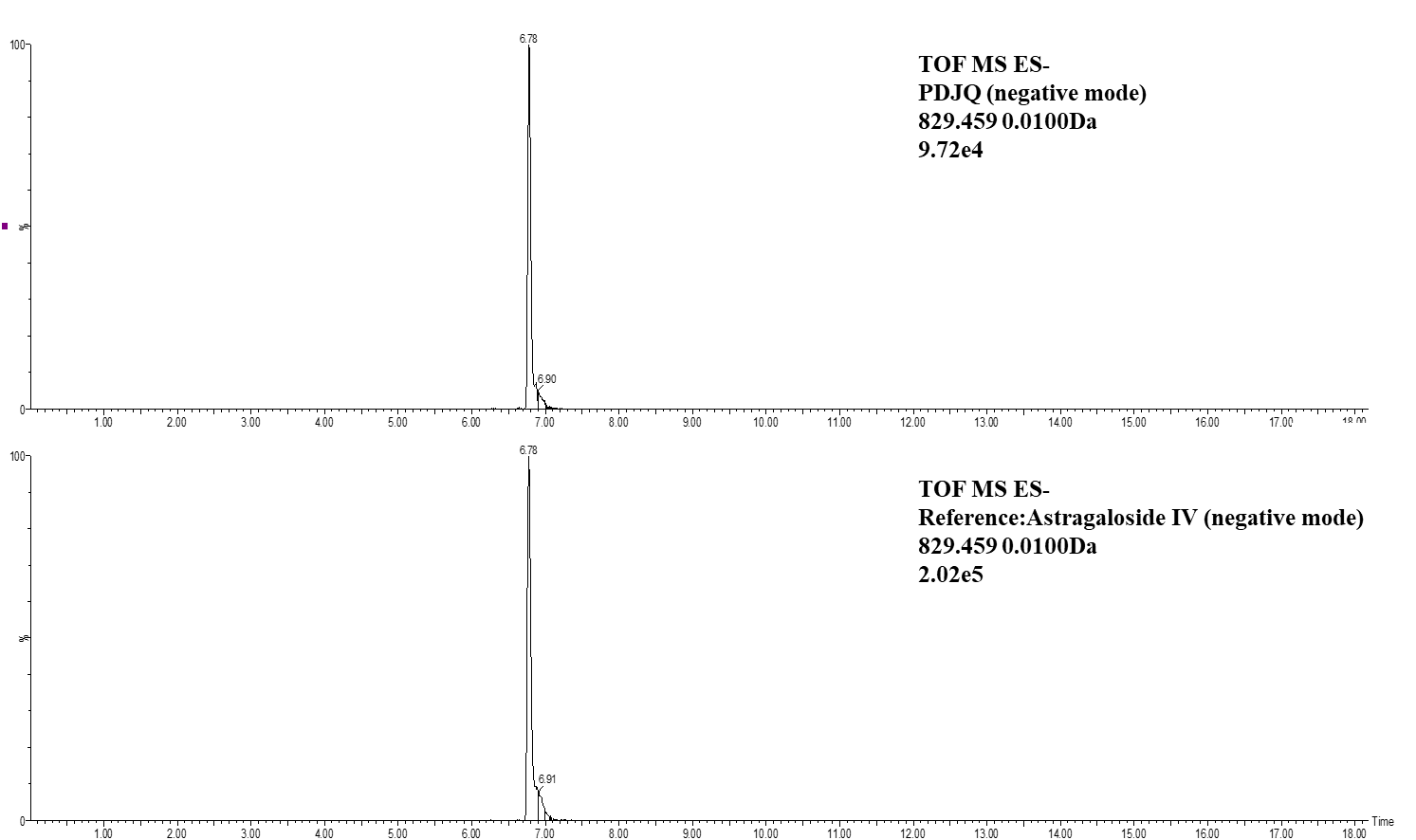
**

**d**

**
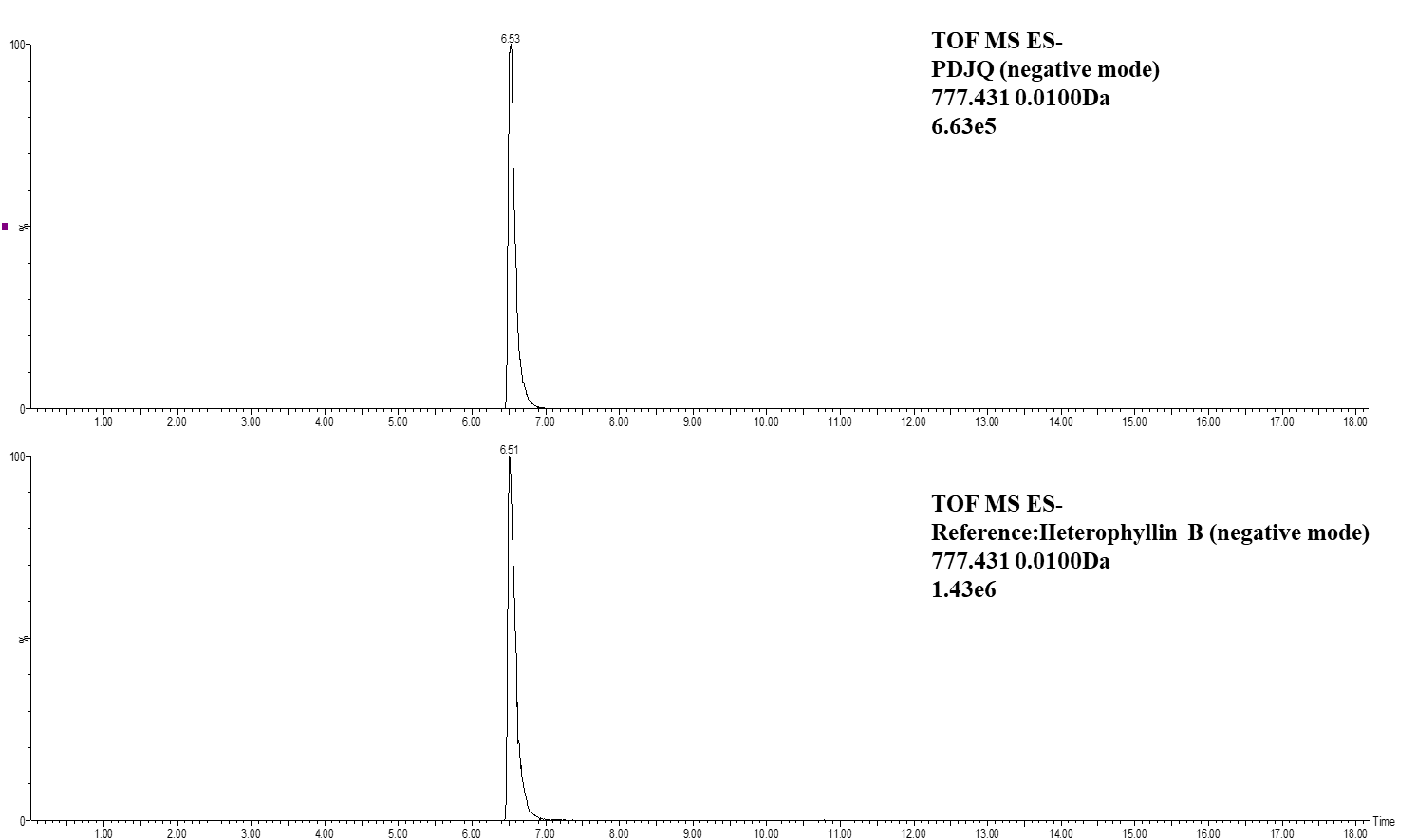
**

**e**

**
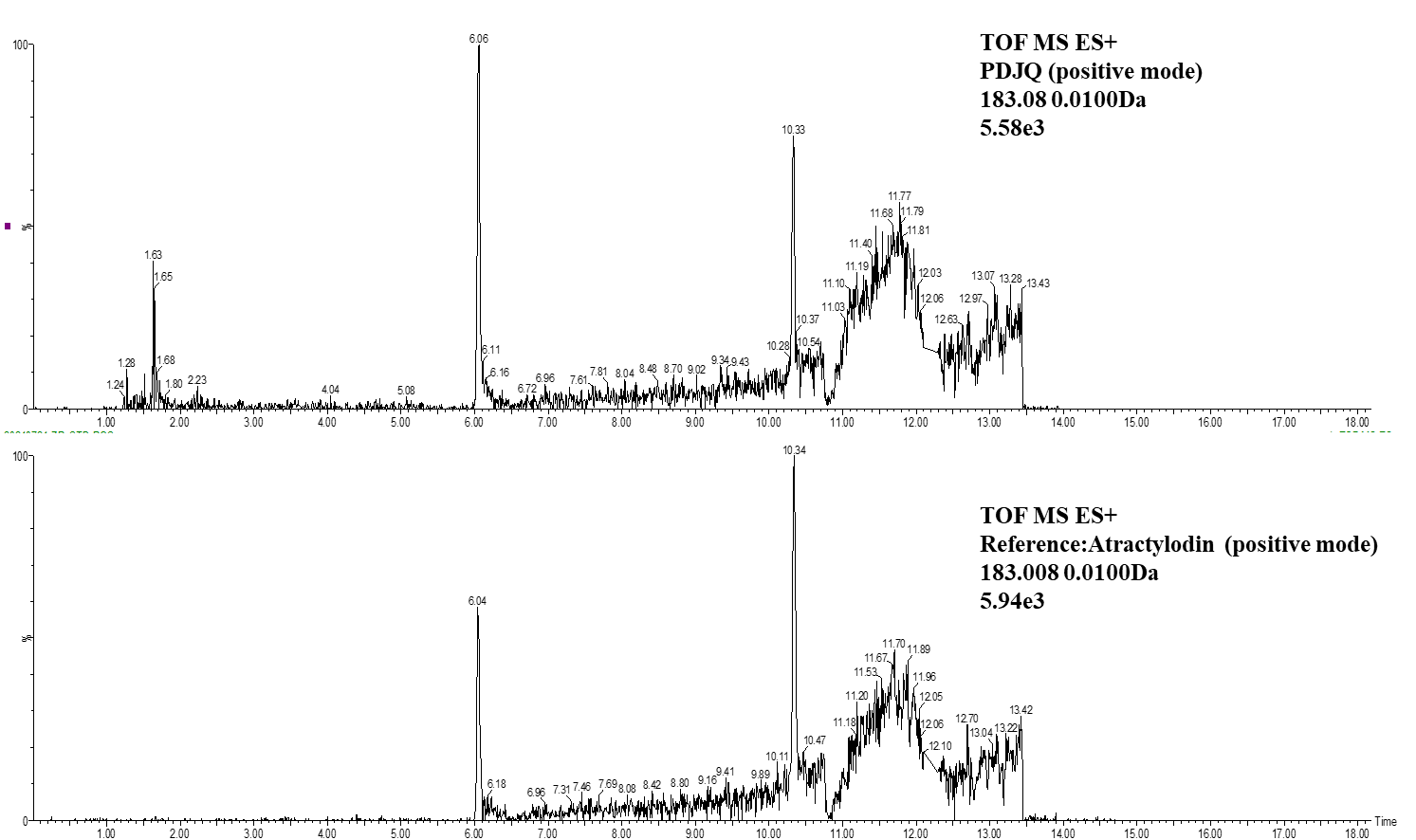
**

**f**

**
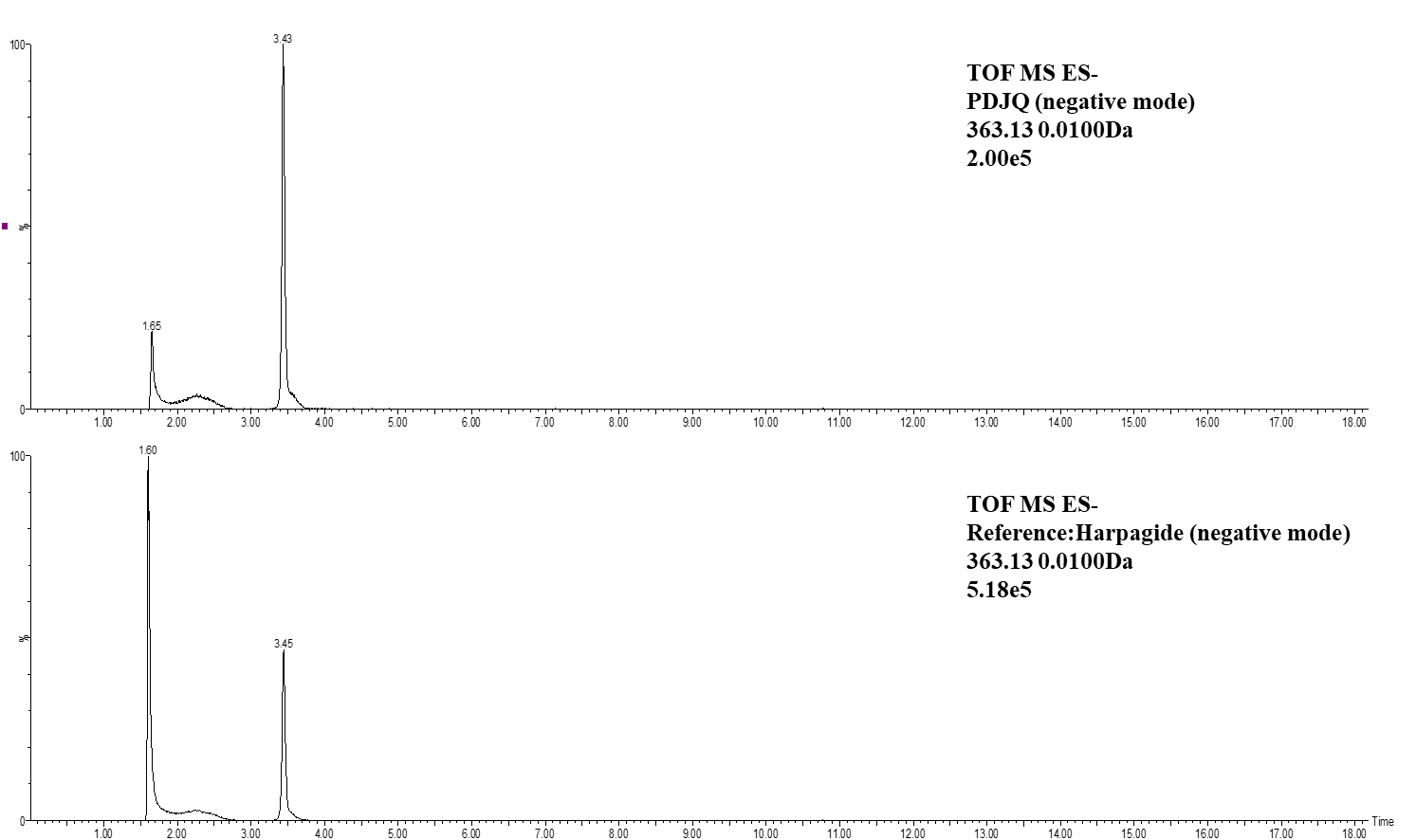
**

**g**

**
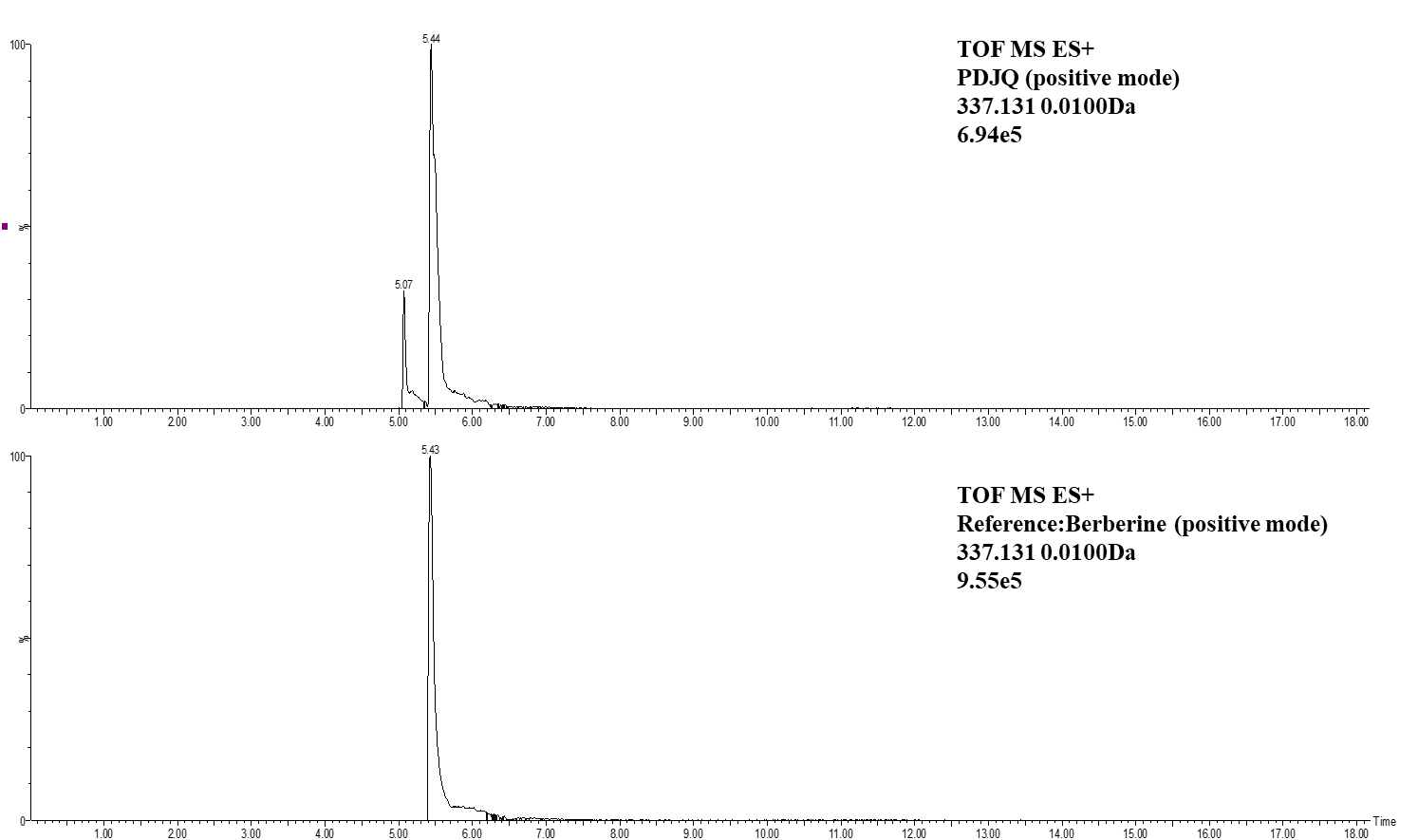
**

**h**

**
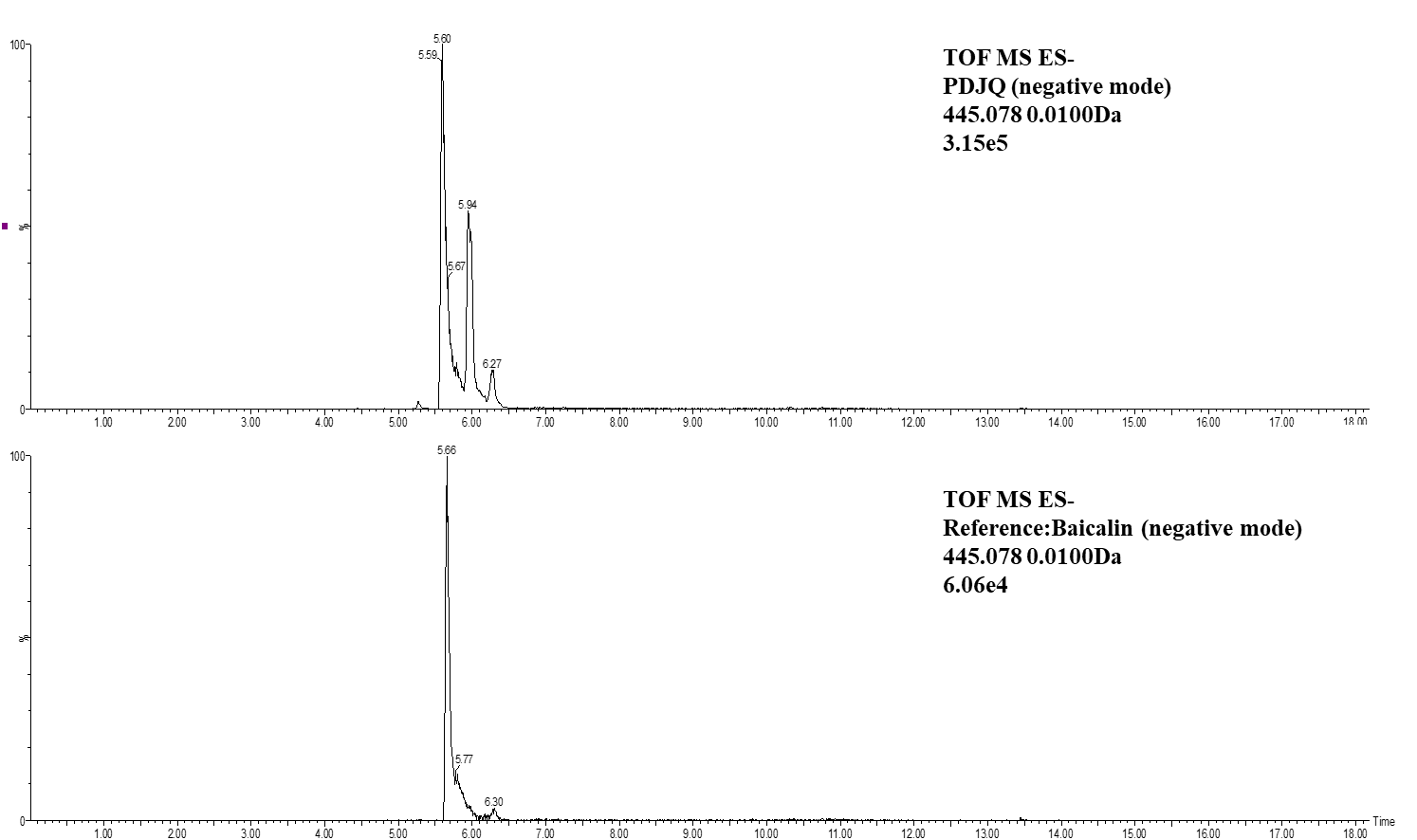
**

**i**

**
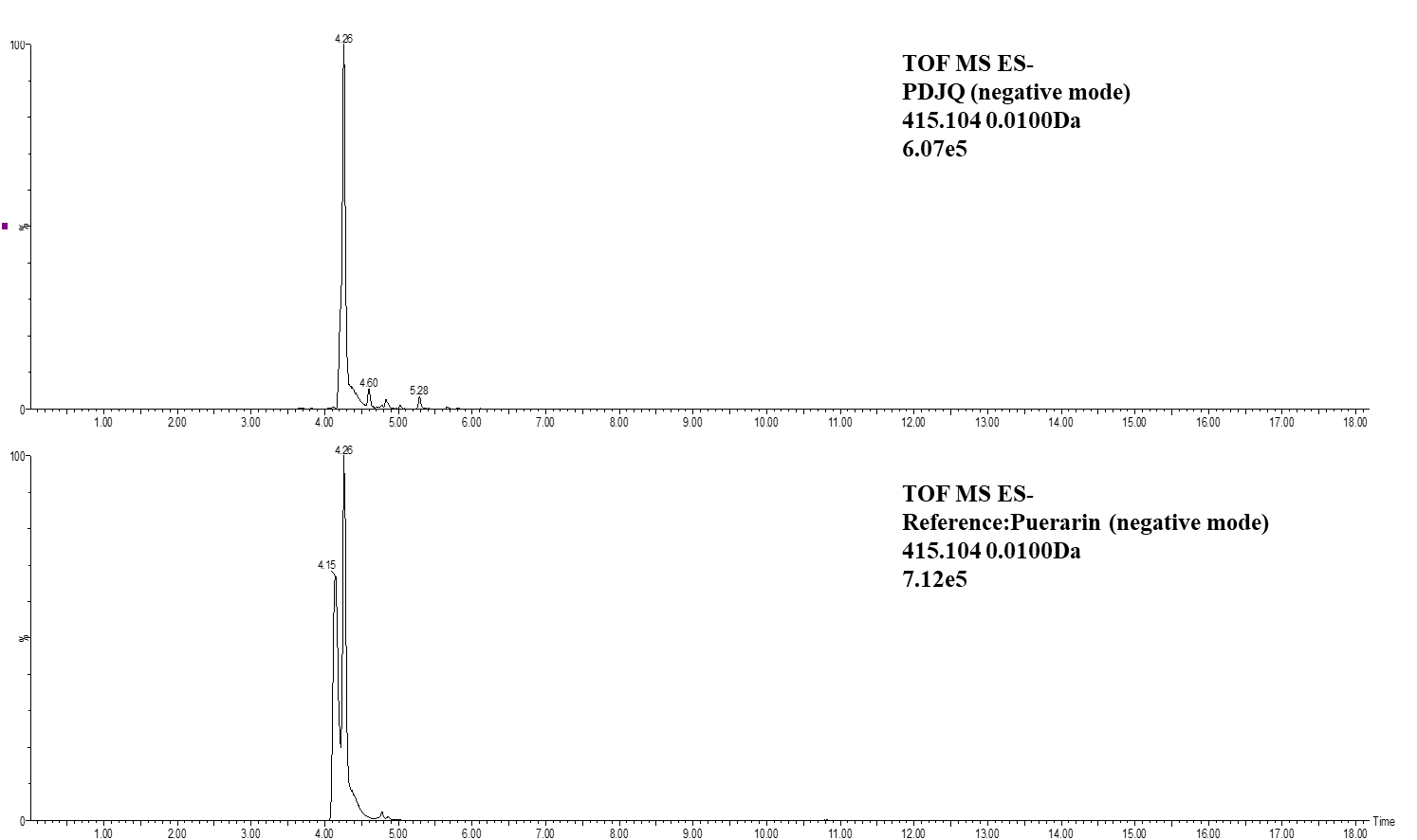
**

**j**

**
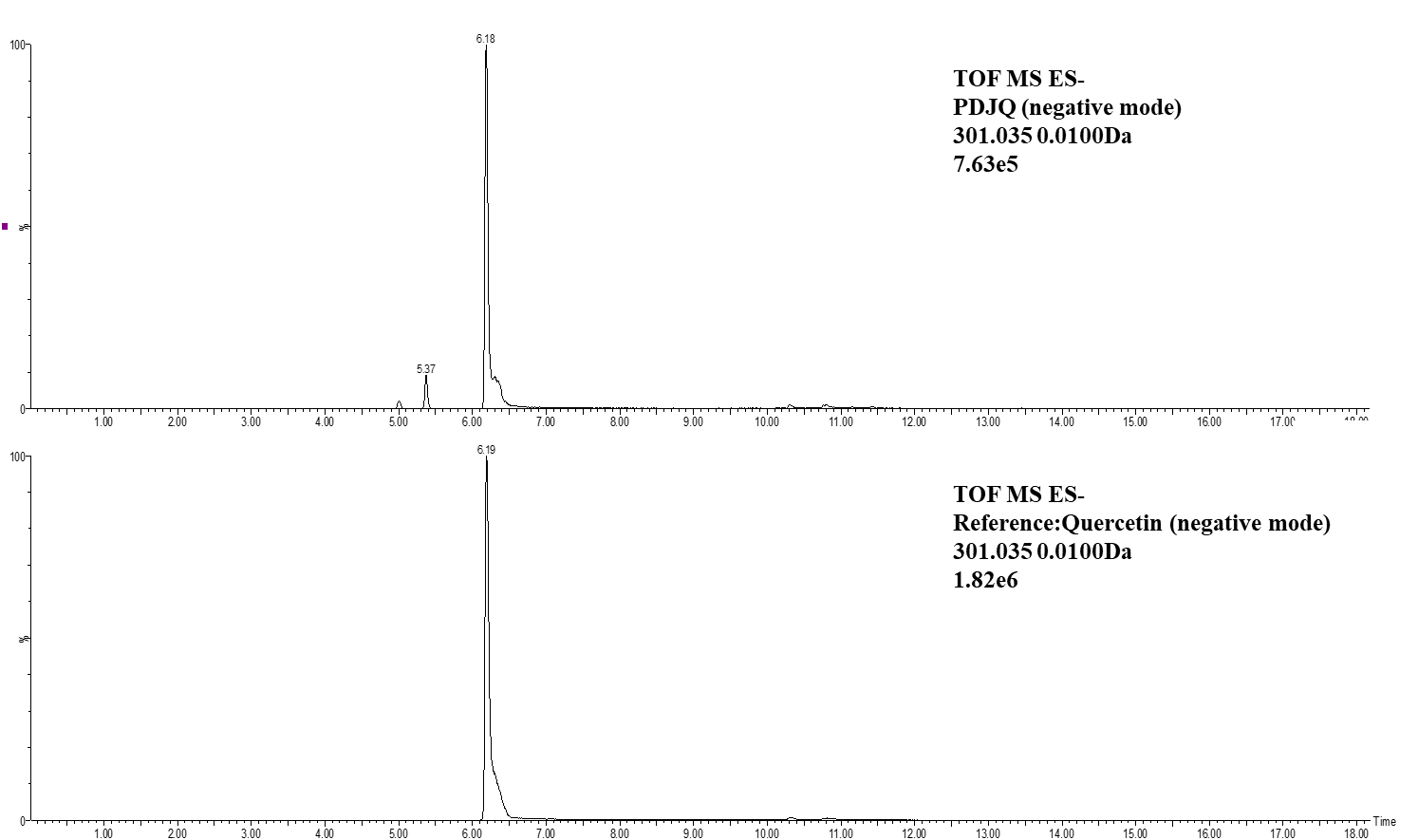
**

**k**

**
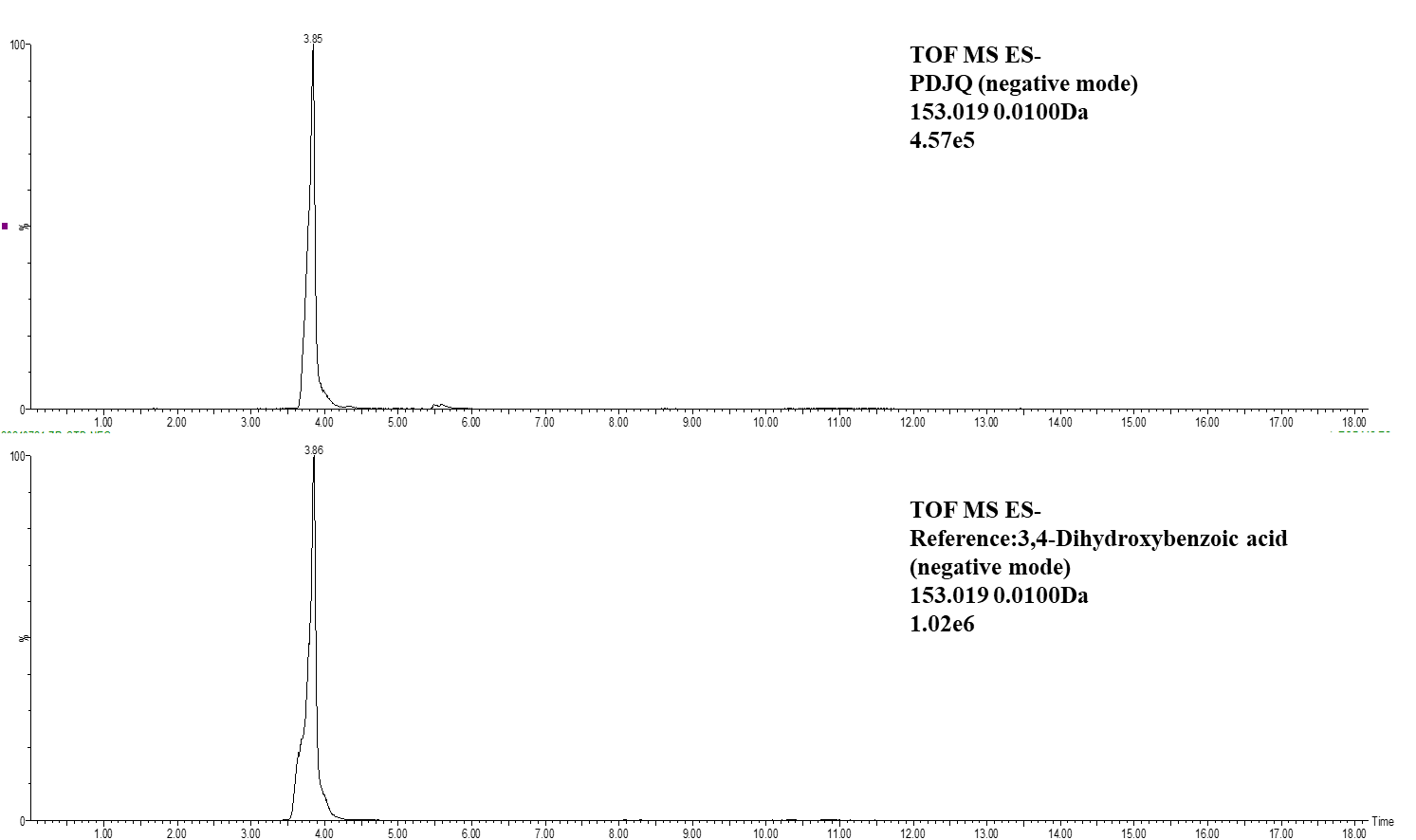
**

**l**

**
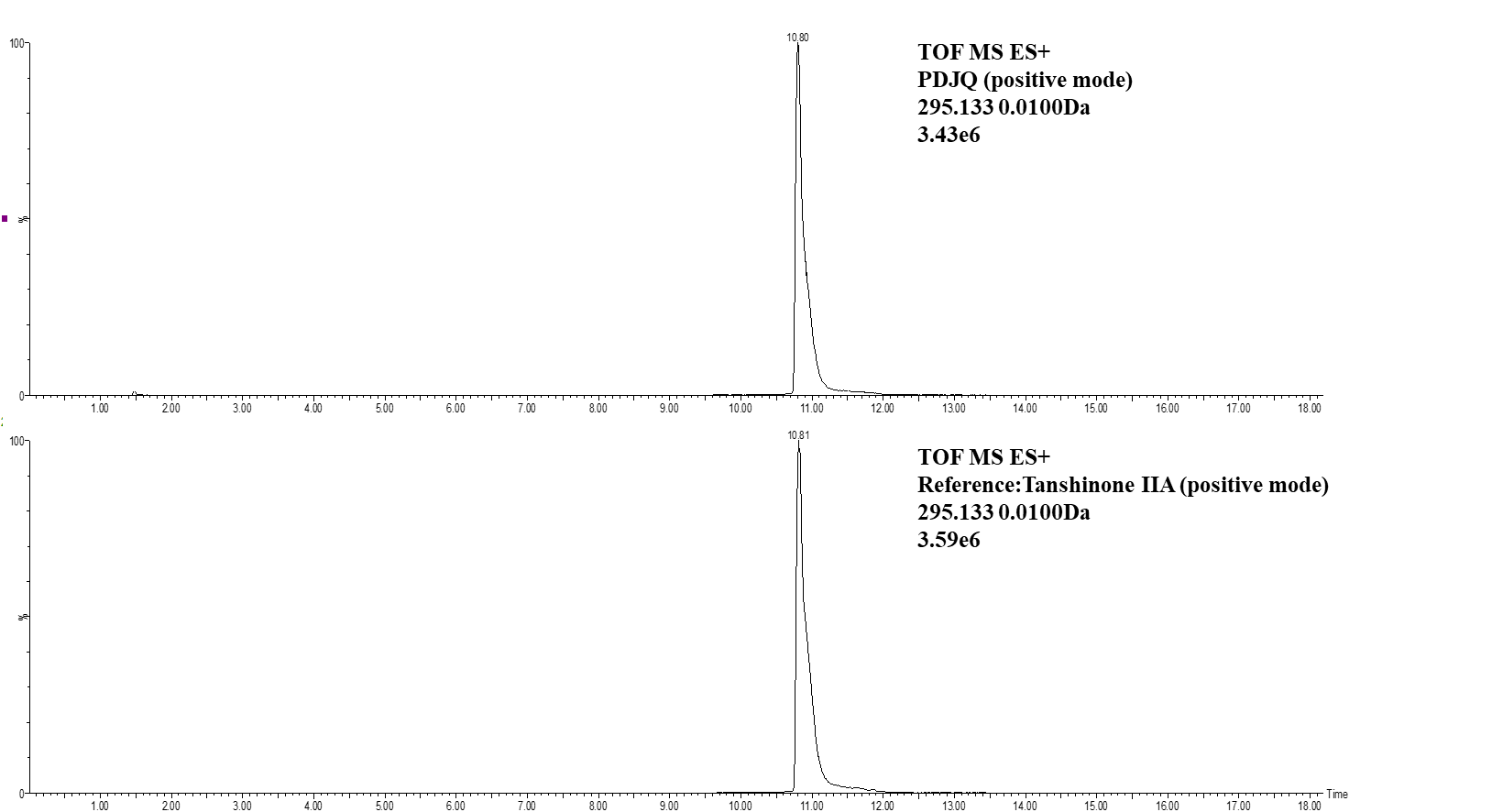
**

**Figure S2:** The chemical profiles of PDJQ using UPLC-MS. (**a, b**) The total ion chromatogram in positive (**a**) and negative ion modes (**b**). (**c-l**) The main bioactive compounds of Astragaloside IV (**c**), Heterophyllin B (**d**), Atractylodin (**e**), Harpagide (**f**), Berberine (**g**), Baicalin (**h**), Puerarin (**i**), Quercetin (**j**), 3,4-Dihydroxybenzoic acid (**k**), Tanshinone IIA (**l**). The bioactive compounds detected in PDJQ were confirmed by the reference standards.

**TABLE S1 The characteristic fragment ions of reference standards in PDJQ**

| **Marking**  **peak no.** | **Name** | **RT**  **(min)** | **Ion** |
| --- | --- | --- | --- |
| 1 | Astragaloside IV | 6.78 | [M-H]^-^ |
| 2 | Heterophyllin B | 6.51 | [M-H]^-^ |
| 3 | Atractylodin | 10.34 | [M+H]^+^ |
| 4 | Harpagide | 3.45 | [M-H]^-^ |
| 5 | Berberine | 5.43 | [M+H]^+^ |
| 6 | Baicalin | 5.66 | [M-H]^-^ |
| 7 | Puerarin | 4.26 | [M-H]^-^ |
| 8 | Quercetin | 6.19 | [M-H]^-^ |
| 9 | 3,4-Dihydroxybenzoic acid | 3.86 | [M-H]^-^ |
| 10 | Tanshinone IIA | 10.81 | [M+H]^+^ |

**TABLE S2 Levels in blood lipid levels in rats selected for 16S rRNA sequencing and metabolomics analysis**

| **Group** | **TC (mmol/L)** | **TG (mmol/L)** | **HDL (mmol/L)** | **LDL (mmol/L)** |
| --- | --- | --- | --- | --- |
| Control | 13.3±5.0 | 2.4±0.4 | 9.2±2.3 | 1.7±0.3 |
| T2DM | 30.2±14.7^##^ | 6.2±1.8^##^ | 4.4±2.9^#^ | 4.3±1.5^##^ |
| PDJQ high-dose | 14.4±3.6^**^ | 2.9±0.9^**^ | 8.5±1.9^**^ | 1.8±0.7^**^ |

Control, T2DM and PDJQ high-dose (n = 6 per group) groups. Data are presented as the mean ± SD. ^#^: *p* < 0.05 as compared to the control group; ^##^: *p* < 0.01 as compared to the control group; ^**^: *p* < 0.01 as compared to the T2DM group

TG: triglyceride; TC: total cholesterol; HDL: high density lipoprotein; LDL: low density lipoprotein; T2DM: type 2 diabetes mellitus; PDJQ: Pi-Dan-Jian-Qing decoction

**TABLE S3 Levels of serum AST, ALT activities and serum Cr, BUN in rats selected for 16S rRNA sequencing and metabolomics analysis**

| **Group** | **AST (U/L)** | **ALT (U/L)** | **Cr (μmol/L)** | **BUN (mmol/L)** |
| --- | --- | --- | --- | --- |
| Control | 94.9±65.0 | 26.8±5.8 | 37.6±14.0 | 3.4±0.4 |
| T2DM | 238.0±74.2^##^ | 78.5±34.3^##^ | 90.7±12.0^##^ | 7.8±2.1^##^ |
| PDJQ high-dose | 103.2±30.4^**^ | 29.0±10.0^**^ | 56.4±13.5^**^ | 5.4±1.9^**^ |

Control, T2DM and PDJQ high-dose (n = 6 per group) groups. Data are presented as the mean ± SD. ^##^: *p* < 0.01 as compared to the control group; ^**^: *p* < 0.01 as compared to the T2DM group

AST: aspartate aminotransferase; ALT: alanine aminotransferase; Cr: creatinine; BUN: blood urea nitrogen

**TABLE S4 Levels of FINS and HOMA-IR in rats selected for 16S rRNA sequencing and metabolomics analysis**

| **Group** | **FINS (μ IU/mL)** | **HOMA-IR** |
| --- | --- | --- |
| Control | 7.2±3.4 | 1.8±0.9 |
| T2DM | 13.4±3.6^##^ | 18.6±6.6^##^ |
| PDJQ high-dose | 8.5±3.1^*^ | 5.1±2.1^**^ |

Control, T2DM and PDJQ high-dose (n = 6 per group) groups. Data are presented as the mean ± SD. ^##^: *p* < 0.01 as compared to the control group; ^*^: *p* < 0.05 as compared to the T2DM group; ^**^: *p* < 0.01 as compared to the T2DM group

HOMA-IR: homeostatic model assessment of insulin resistance; FINS: fasting insulin

**a**


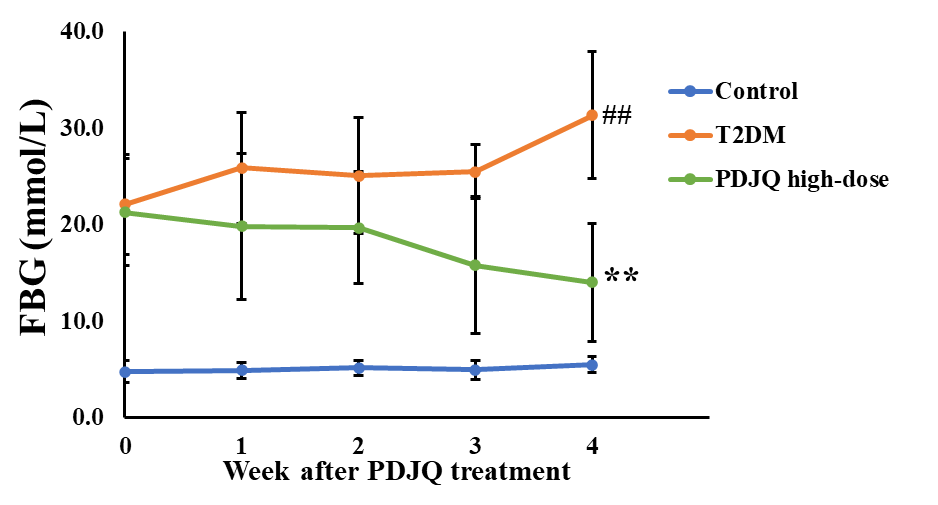


**b**


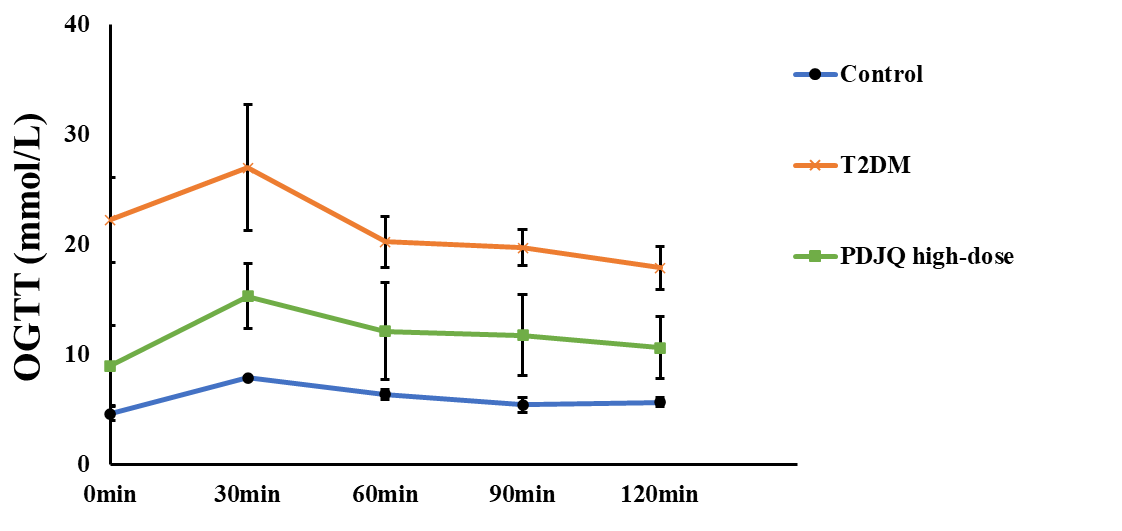


**c**

**
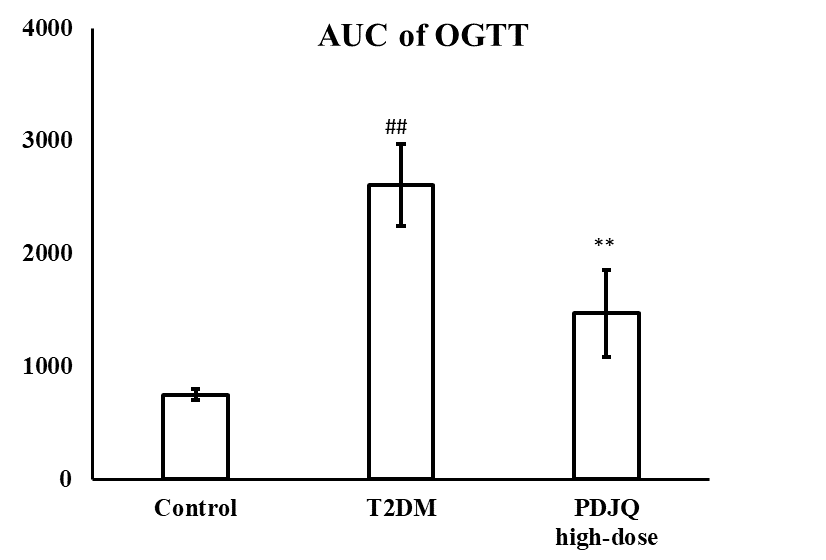
**

**Figure S3** Levels of FBG and AUC of OGTT in rats selected for 16S rRNA sequencing and metabolomics analysis. (**a**) Rats selected for 16S rRNA sequencing and metabolomics analysis showed statistical significances in FBG between Control and T2DM groups and between T2DM and PDJQ high-dose groups. (**b,c**) Rats selected for 16S rRNA sequencing and metabolomics analysis showed statistical significances in the AUC of OGTT between Control and T2DM groups and between T2DM and PDJQ high-dose groups.

Control, T2DM and PDJQ high-dose (n = 6 per group) groups. Data are presented as the mean ± SD. ^##^: *p* < 0.01 as compared to the control group; ^**^: *p* < 0.01 as compared to the T2DM group
